# Glucose Transporter 1 in Health and Disease

**DOI:** 10.1002/mco2.70874

**Published:** 2026-07-22

**Authors:** Yi Tai, Zhihao Chen, Yuping Zhu, Weidong Han

**Affiliations:** ^1^ Department of Colorectal Surgery Zhejiang Cancer Hospital Hangzhou China; ^2^ Hangzhou Institute of Medicine (HIM) Chinese Academy of Sciences Hangzhou China; ^3^ Department of Musculoskeletal Oncology State Key Laboratory of Oncology in South China Collaborative Innovation Center for Cancer Medicine Guangdong Provincial Clinical Research Center for Cancer Sun Yat‐sen University Cancer Center Guangzhou PR China; ^4^ Department of Colorectal Medical Oncology Zhejiang Cancer Hospital Hangzhou China

**Keywords:** glucose homeostasis, glucose transporter 1 (GLUT1), tumor microenvironment (TME), targeted therapy, Warburg effect

## Abstract

Glucose Transporter 1 (GLUT1) is the quintessential facilitator of basal glucose uptake, indispensable for maintaining cellular energy homeostasis, particularly across the blood–brain barrier. Beyond physiological necessity, GLUT1 dysregulation drives a broad pathological spectrum. While genetic haploinsufficiency precipitates severe neurological energy crises like Glut1 deficiency syndrome (Glut1DS), oncogenic networks hyperactivate GLUT1 as the central executor of the Warburg effect to fuel malignant proliferation. Despite its immense therapeutic potential, severe on‐target toxicity in normal tissues and adaptive metabolic plasticity remain critical roadblocks to systemic GLUT1 inhibition. This review comprehensively synthesizes GLUT1's multidimensional regulatory networks in health and disease, dissecting how its overexpression fundamentally remodels the tumor microenvironment (TME). We elucidate how GLUT1‐driven “metabolic competition” fosters metabolic immune exclusion and drives therapeutic resistance. Furthermore, we map the paradigm shift from traditional systemic blockades to emerging precision interventions. Specifically, we highlight “Trojan horse” glycan‐functionalized nanocarriers, targeted protein degradation technologies like PROTACs, and metabolically engineered CAR‐T cells. By conceptualizing GLUT1 as the linchpin of the immunosuppressive ecosystem, this work provides a strategic roadmap for precision metabolic immuno‐oncology, guiding the development of novel therapies that maximize durable efficacy while minimizing collateral physiological damage.

## Graphical abstract

As the quintessential facilitator of basal glucose uptake, glucose transporter 1 (GLUT1) is indispensable for maintaining systemic energy homeostasis. This graphical abstract delineates the multidimensional landscape of GLUT1 in normal physiology. It highlights its tissue‐specific metabolic roles—from fueling erythrocytes and fetal development to supporting astrocyte‐mediated brain homeostasis. Furthermore, it maps the intricate regulatory networks governing GLUT1 expression and function, encompassing transcriptional control (e.g., HIF‐1α), epigenetic modifications, signaling pathways (PI3K/Akt/mTOR), and dynamic membrane trafficking (e.g., TXNIP‐mediated endocytosis). Crucially, the schematic underscores GLUT1's pivotal role in immunometabolism, illustrating how its tightly regulated glucose flux drives the metabolic reprogramming, polarization, and inflammatory responses of immune cells such as macrophages and T‐cells.

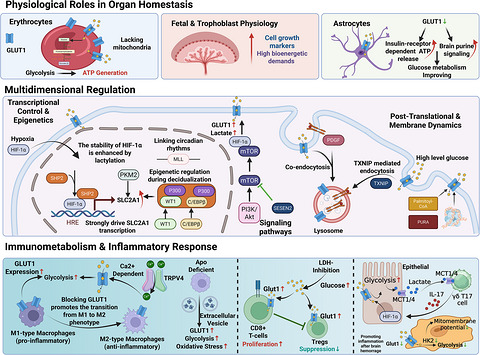

## 1 Introduction

The history and discovery of glucose transporter type 1 (GLUT1), a facilitative glucose transporter protein, are intrinsically linked to the broader scientific quest to understand cellular energetics. The fundamental concept of glucose transport across cell membranes has been a subject of inquiry for decades, evolving from the realization that cells require a constant supply of their primary energy source to the identification of specific proteins mediating this uptake. The initial proposal of the Na+‐glucose cotransport hypothesis by Crane in 1960 marked a pivotal moment in this timeline [1]. Since the subsequent cloning of SGLT1 in 1987, significant advancements have been made in characterizing the genetics, molecular biology, and biophysics of glucose transporters [1]. The discovery of facilitative glucose transporters (GLUTs) further clarified how glucose traverses hydrophobic cellular barriers without direct energy expenditure, driven solely by concentration gradients [2, 3]. The solute carrier family 2 (SLC2A) genes encode these facilitative glucose transporters, a family comprising 14 distinct isoforms (GLUT1–14) that play a crucial role in mediating glucose movement across cell membranes [4, 5, 6]. Within this family, there is a clear evolutionary division of labor tailored to diverse physiological needs: GLUT2, for example, acts as a low‐affinity glucose sensor in hepatocytes and pancreatic β‐cells [7], while GLUT4 serves as the primary insulin‐responsive transporter in muscle and adipose tissues, critical for postprandial glucose disposal [8]. Standing apart from these specialized isoforms is GLUT1, encoded by the SLC2A1 gene. As the primary mediator of basal, non‐insulin‐mediated glucose uptake, GLUT1 is a quintessential “housekeeping” protein essential for cellular survival and function across a wide array of mammalian cells [9]. In normal physiology, GLUT1 demonstrates a distinctive tissue distribution pattern, with particularly high expression in specialized barrier tissues such as the blood–brain barrier (BBB) endothelial cells—where it accounts for over 90% of glucose transporters—as well as in erythrocytes, placental trophoblasts, and renal proximal tubules [9]. Its high affinity for glucose enables effective uptake even at low extracellular concentrations [10], a kinetic property essential for tissues with continuous energy demands. In endothelial cells, GLUT1‐mediated glycolysis generates ATP for cellular maintenance [11], while in the central nervous system (CNS), it is central to maintaining BBB integrity and facilitating neural glycolysis [12].

However, glucose homeostasis is vital for maintaining overall health, and the dysregulation of GLUT1 function is inextricably linked to a spectrum of pathological conditions ranging from rare genetic disorders to widespread metabolic diseases [13, 14]. In recent years, the role of GLUT1 has received extensive attention beyond its canonical metabolic functions. For instance, Glut1DS, a rare genetic disorder characterized by impaired glucose transport into the brain, underscores the transporter's nonredundant role in neurological development and survival [15]. Beyond the nervous system, GLUT1 is critical in regulating the metabolic reprogramming of immune cells. In sepsis‐induced lung injury, macrophages rely on GLUT1‐mediated glycolysis for effective phagosome maturation [16], while in chronic inflammation and autoimmune conditions such as diabetic osteoarthritis, aberrant GLUT1 expression drives pathogenic stromal and immune responses [17, 18, 19]. Thus, GLUT1 serves as a nexus linking basal metabolism, immune regulation, and systemic homeostasis. It is this foundational role in sustaining cellular life that is arguably most perversely hijacked during malignant transformation. The transition from a regulated physiological facilitator to a pathological driver represents a hallmark of cancer metabolic reprogramming. In this context, GLUT1 emerges as the central executor of the Warburg effect—the paradoxical preference of cancer cells for aerobic glycolysis over oxidative phosphorylation, even under oxygen‐replete conditions [20, 21]. This metabolic rewiring is essential for supporting rapid tumor cell proliferation and survival [22], particularly within the hypoxic and nutrient‐deprived TME [23]. By mediating enhanced glucose uptake, GLUT1 enables cancer cells to maintain a glycolytic phenotype that provides not only ATP but also the crucial biosynthetic precursors necessary for rapid biomass accumulation. This pathological upregulation is actively driven by a convergence of oncogenic signaling and environmental cues. The TME, characterized by hypoxia and extracellular acidosis, exerts multifaceted control over GLUT1. HIF‐1α serves as the primary transcriptional regulator, becoming stabilized under low‐oxygen conditions to bind hypoxia‐responsive elements (HREs) and potently drive SLC2A1 transcription [24, 25]. Beyond hypoxia, this metabolic shift is reinforced by cell‐intrinsic oncogenic mutations, such as those in IDH1/2, which upregulate GLUT1 via the PI3K/Akt/mTORC1 signaling axis, enabling tumor cells to dynamically adapt to environmental stress [26]. Emerging evidence also implicates systemic neuroendocrine signaling; for example, sympathetic nervous system‐derived norepinephrine creates a metabolically permissive niche by upregulating GLUT1 [27]. In glioblastoma, this hypoxia‐induced metabolic plasticity confers profound adaptability, allowing cells to sustain energy homeostasis when canonical pathways are disrupted [24, 28].

Crucially, GLUT1's influence extends far beyond metabolic sustenance; it is an active orchestrator of tumor aggressiveness and TME remodeling. Elevated GLUT1 expression is mechanistically linked to enhanced invasiveness and metastatic potential, as evidenced by its ability to upregulate matrix metalloproteinase‐2 (MMP‐2) for extracellular matrix degradation [29]. In non–small cell lung cancer (NSCLC) and pancreatic cancer, GLUT1 overexpression correlates with accelerated progression, metastatic propensity, and increased ^1^ ^8^F‐FDG uptake [20, 21, 30]. Furthermore, GLUT1 fuels the proliferation of malignant cells, correlating with markers such as Ki67 [31], and maintains leukemic stem cell plasticity in acute myeloid leukemia (AML) [32]. Within the TME, GLUT1 promotes metabolic crosstalk with stromal components, including cancer‐associated fibroblasts (CAFs) and tumor‐associated macrophages (TAMs), fostering an immunosuppressive niche that promotes therapeutic resistance [33]. The profound biological roles of GLUT1 are directly reflected in its clinical utility. Elevated expression correlates strongly with aggressive phenotypes and poor prognosis across multiple malignancies, including colorectal, breast, gastric, and hepatocellular carcinomas [26, 34, 35]. Meta‐analyses consistently demonstrate that high GLUT1 levels are associated with reduced overall survival (OS) and disease‐free survival (DFS) [34, 35], making it a robust prognostic biomarker and a potential predictor of therapeutic response, as seen in melanoma [36]. Despite its validation as a high‐value target, directly inhibiting GLUT1 is complicated by the inherent metabolic plasticity of tumors, which often activate compensatory pathways [37, 38]. Consequently, a new generation of therapeutic strategies is being developed, ranging from direct inhibitors (e.g., BAY‐876) and indirect suppression via upstream regulators (e.g., HIF‐1α inhibitors) to innovative nanocarriers and PROTAC degraders [39, 40]. By disrupting GLUT1‐mediated glucose metabolism and its interconnected signaling networks, these innovations, particularly when used in rational combination therapies [2, 39], hold significant promise for enhancing treatment efficacy, overcoming resistance, and ultimately improving clinical outcomes for cancer patients [21, 41].

In this review, we provide a comprehensive overview of GLUT1's role across the spectrum of health and disease to offer new insights for precision medicine. We first delineate the structural basis and physiological regulation of GLUT1 as the primary mammalian glucose transporter. Subsequently, we examine its pathogenic role in nonmalignant disorders, including Glut1DS, diabetes, and inflammatory conditions. Finally, we provide a comprehensive analysis of GLUT1 as a central driver in tumorigenesis, microenvironment remodeling, and therapeutic resistance, highlighting emerging strategies for targeting this metabolic nexus.

## 2 GLUT1 in Normal Physiology

Before examining the pathological hijacking of GLUT1, it is imperative to establish its foundational role in maintaining basal mammalian physiology. As a highly conserved member of the SLC2A family, GLUT1 distinguishes itself through a unique molecular architecture tailored for constitutive, high‐affinity glucose transport. This metabolic conduit is strictly compartmentalized yet universally essential, serving as the nonredundant gatekeeper for continuous energy supply across specialized boundaries—most notably the BBB and erythrocyte membranes—while critically supporting early embryogenesis. Beyond these classical bioenergetic paradigms, GLUT1 has recently emerged as a pivotal orchestrator of immunometabolism, fundamentally dictating immune cell polarization and driving inflammatory responses. To fulfill these diverse functional demands without perturbing systemic homeostasis, GLUT1 expression and membrane dynamics are tightly calibrated by a sophisticated, multidimensional regulatory network. By delineating the interplay between its foundational transport functions, immunological roles, and the precise transcriptional and posttranslational safeguards that govern its activity, this section provides the crucial mechanistic context required to fully comprehend its dysregulation in subsequent disease and malignancy.

### 2.1 Mechanisms of Glucose Homeostasis and the SLC2A Family Landscape

Glucose homeostasis is a fundamental prerequisite for mammalian life, requiring the precise orchestration of glucose entry into cells across the hydrophobic lipid bilayer of the plasma membrane. This process is mediated by two distinct classes of transport proteins. The first class comprises the active transporters or sodium‐glucose cotransporters (SGLTs), encoded by the SLC5 gene family. These transporters concentrate glucose inside cells using the transmembrane electrochemical potential of sodium (Na^+^). The SGLT family includes 12 members that function as symporters, cotransporting not only sugars but also anions, vitamins, and short‐chain fatty acids [1, 42]. While often associated with the kidney, their role extends to other tissues; for instance, glucose transport across the BRB relies on both SGLTs (SGLT1 and SMIT1) and GLUTs to nourish the neuronal retina [43]. Similarly, in the heart, SGLT‐mediated uptake may be linked to intracellular signaling rather than direct energy production [44]. However, the majority of mammalian cells import glucose through a process mediated by the second class: facilitated transporters (GLUTs). This passive process is governed by the solute carrier family 2 (SLC2A) genes, which encode 14 distinct facilitative glucose transporter isoforms (GLUT1–14) that play a crucial role in mediating glucose movement across cell membranes [1, 42, 45, 46].

Within the SLC2A family, there is a clear evolutionary division of labor tailored to diverse physiological needs. GLUT2 (*SLC2A2*) is a high‐capacity, low‐affinity isoform expressed in hepatocytes, pancreatic β‐cells, the small intestine, and the central nervous system. It allows uninhibited flux of glucose to balance concentrations across the cellular membrane, and its variations are linked to various endocrine and metabolic disorders [14, 45]. In contrast, GLUT4 (*SLC2A4*) is expressed exclusively in insulin‐sensitive tissues such as fat and muscle, responsible for postprandial glucose disposal under the modulation of insulin and catecholamines [8, 45, 47]. Other members exhibit unique specificities: GLUT3 (*SLC2A3*) is a low‐Km isoform responsible for glucose uptake into neurons [6, 45]; GLUT5 (*SLC2A5*) is abundant in spermatozoa and intestinal cells as a fructose transporter [45]; and GLUT7 (*SLC2A7*) facilitates the flux of free glucose out of the endoplasmic reticulum [45]. Notably, some members transport substrates other than glucose; for example, SLC2A9 (GLUT9) is a major determinant of plasma uric acid levels, linking it to gout development [46, 48].

**FIGURE 1:**
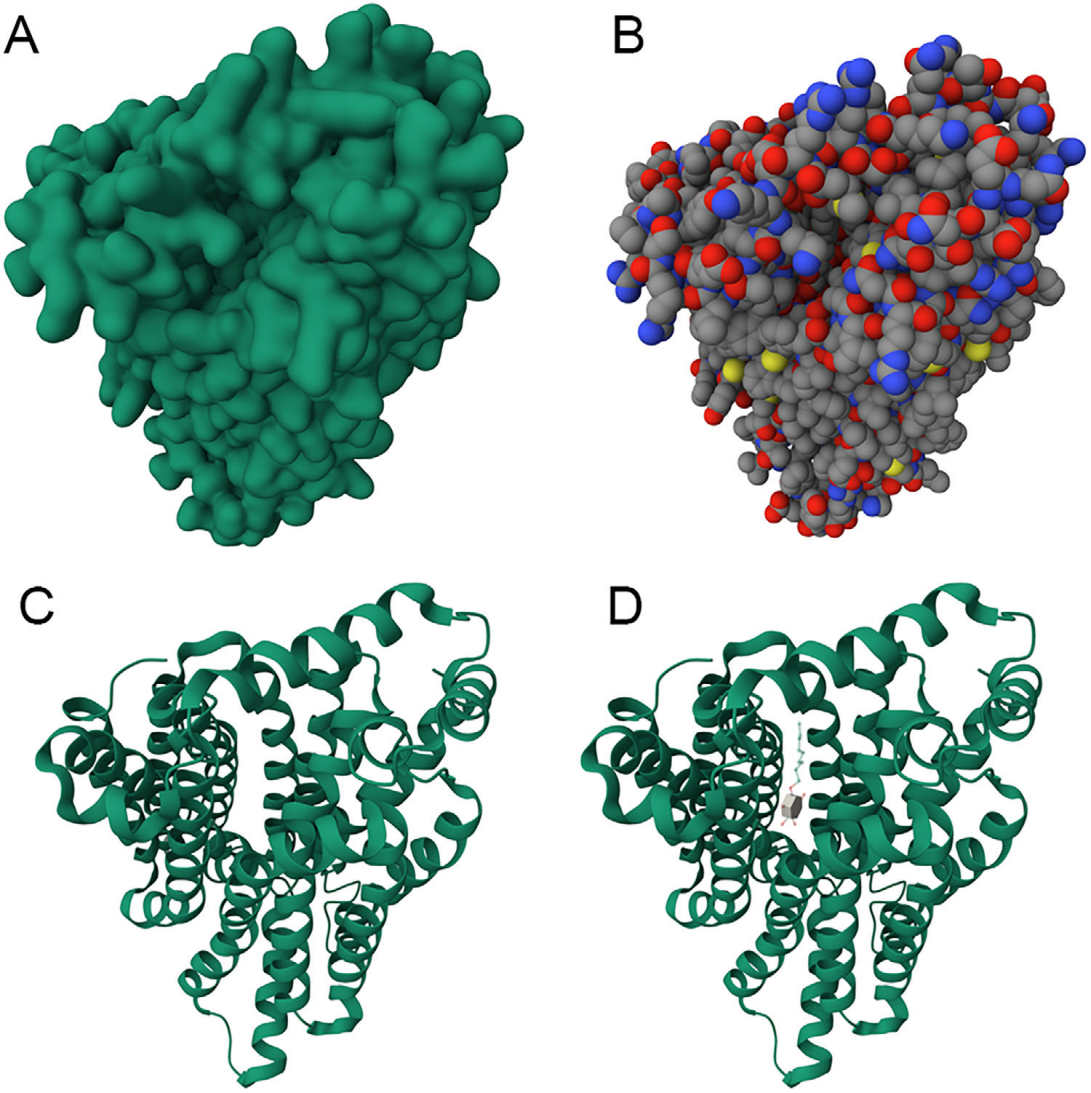
Multiscale structural models of the GLUT1 transporter. The architecture of GLUT1 is depicted through (A) a coarse‐grained Gaussian surface representation, (B) an all‐atom structural model, and (C) a protein backbone conformation. (D) GLUT1 in complex with its ligand, nonyl‐beta‐d‐glucopyranoside.

Amidst this diversity, glucose transporter 1 (GLUT1, encoded by SLC2A1) stands apart as the widely expressed isoform that provides many cells with their basal glucose requirement [45]. Its functional properties are rooted in its molecular architecture. As illustrated in Figure 1, crystallographic studies have shown that GLUT1 presents typical transmembrane protein folding characteristics, containing 12 transmembrane α‐helices (TMHs) that form a water‐filled channel to accommodate the passage of glucose molecules [49, 50]. Mechanistically, GLUT1 operates as a uniporter via an alternating access mechanism, where its conformation cyclically changes to expose a glucose‐binding site to the outside and then the inside of the cell, allowing glucose to flow down its concentration gradient [1, 49]. This high‐affinity transporter is essential for tissues with high and continuous energy demands, distinguishing it as a quintessential “housekeeping” protein [9, 10]

### 2.2 Physiological Roles in Organ Homeostasis and Development

**FIGURE 2:**
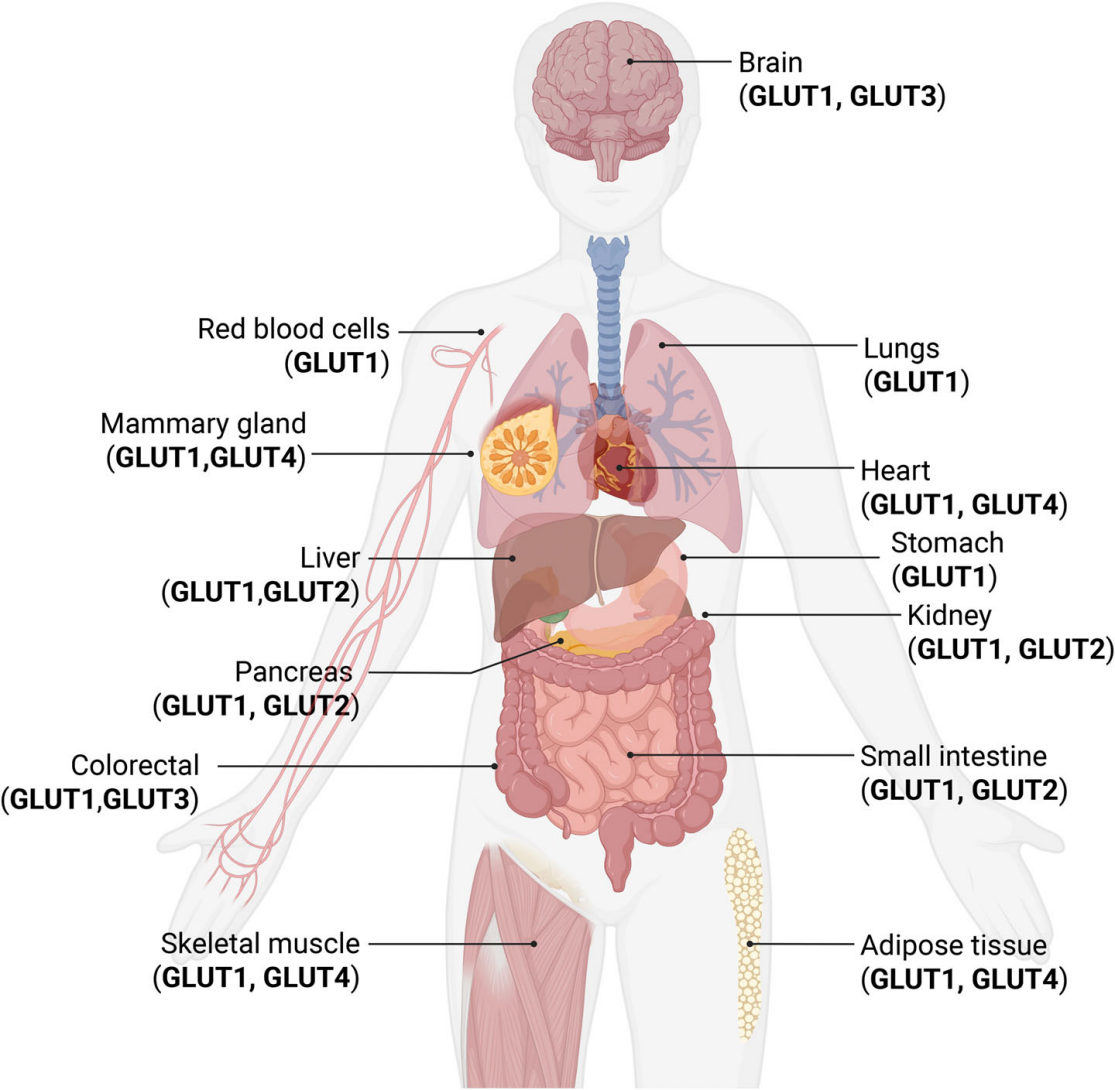
Differential expression profile of glucose transporter (GLUT) isoforms across major human organ systems. While widely expressed, GLUT1 is uniquely enriched in specialized barrier tissues (such as the blood‐brain barrier) and high‐metabolism organs. This strategic distribution ensures continuous basal glucose uptake to meet the strict bioenergetic demands of these distinct physiological compartments. *Source*: Created with bioRender.com, with permission.

While GLUT1 is widely expressed, its distribution is strategically enriched in specialized barrier tissues and organs with high metabolic rates, as depicted in Figure 2.

#### 2.2.1 The Blood–Brain Barrier and Central Nervous System Energy Supply

The most critical physiological role of GLUT1 is arguably found in the CNS. GLUT1 is prominently expressed in the endothelial cells of the BBB as well as in neuronal membranes (alongside GLUT3), where it plays a special role in transporting glucose across epithelial and endothelial barrier tissues [6, 9]. It accounts for over 90% of the glucose transporters in BBB endothelial cells, acting as the central gatekeeper for maintaining BBB integrity and facilitating neural glycolysis [9, 12].

The indispensable nature of this transport system is illustrated by Glut1DS. Caused by GLUT1 dysfunction, this disorder is characterized by impaired glucose transport into the brain, leading to a spectrum of neurological symptoms including paroxysmal eye‐head movements, pharmacoresistant seizures, deceleration of head growth, developmental delay, and intellectual disability [15, 51]. Current treatments mainly focus on ketogenic diet therapies (KDTs) to provide alternative brain fuel [6, 51]. Interestingly, recent studies suggest a complex role for GLUT1 in brain homeostasis beyond simple transport. In mice, a reduction of astrocytic GLUT1 paradoxically improves central and peripheral glucose metabolism, potentially through increased insulin receptor‐dependent ATP release and brain purine signaling [52].

#### 2.2.2 Fetal and Erythroid Physiology

In the hematopoietic system, GLUT1 is highly expressed on the membrane of erythrocytes [9]. Lacking mitochondria, red blood cells rely exclusively on glycolysis for their energy needs, making them completely dependent on a continuous supply of glucose facilitated by GLUT1 [49, 53].

Furthermore, GLUT1 is crucial for prenatal development. It is highly expressed in the placenta, specifically in trophoblasts, where it mediates glucose uptake into the fetus [9, 54]. The association of GLUT1 with high‐proliferation contexts is further evidenced by its correlation with cell growth markers, linking it to the high bioenergetic demands of embryonic development [55, 56].

### 2.3 Immunometabolism And Inflammatory Responses

In addition to its role in basal metabolism, GLUT1 is a central regulator of immune cell function and inflammation. The metabolic reprogramming of immune cells—often involving a shift toward glycolysis—is heavily dependent on GLUT1 expression.

#### 2.3.1 Macrophage Polarization and Function

GLUT1‐mediated glycolysis is pivotal for macrophage activation. In sepsis‐induced lung injury, macrophages regulate glycolysis by enhancing glucose uptake through GLUT1, a process regulated by TRPV4 channels. This metabolic shift is crucial for effective phagosome maturation and limiting lung injury [16]. The expression level of GLUT1 is closely related to the glycolytic ability of pro‐inflammatory M1 macrophages [57]. For instance, in diabetic osteoarthritis, increased glycolysis in fibroblast‐like synoviocytes drives synovial macrophage infiltration and M1 polarization via the YAP1/TXNIP signaling axis, which regulates GLUT1‐dependent glycolysis [17]. Conversely, inhibiting GLUT1 can promote the transformation of M1 macrophages to the anti‐inflammatory M2 phenotype, suggesting therapeutic potential in inflammatory diseases [58]. Extracellular vesicles derived from macrophages can further modulate inflammation; for instance, vesicles from ApoE‐deficient macrophages increase NF‐κB‐driven, GLUT1‐mediated glycolysis, thereby promoting oxidative stress and inflammation [59]. Even in tumor contexts, such as glioblastoma, monocyte‐derived macrophages exhibit high GLUT1 expression and glycolytic activity, which unfortunately promotes immunosuppressive activity [60].

#### 2.3.2 T‐Cell and Neutrophil Activity

The dependence on glycolysis is also a feature of other immune cells. In T cells, enhancing glucose uptake and GLUT1 expression—for example, by inhibiting lactate dehydrogenase (LDH)—can improve their tumor‐killing function and proliferation while inhibiting the immunosuppressive activity of regulatory T cells (Tregs) [61]. Similarly, neutrophils increase GLUT1 expression under hyperglycemic conditions. This upregulation enhances glucose uptake and glycolysis, leading to the release of neutrophil extracellular traps (NETs), which drive inflammation and barrier disruption in mucosal tissues [62]. Additionally, GLUT1‐dependent glucose metabolism helps maintain the expression of surface markers like CD115 on monocytes and regulates their migratory ability by modulating CCR2 expression [63].

#### 2.3.3 Tissue‐Specific Inflammation

Beyond immune cells, GLUT1 regulates inflammatory responses in tissue cells. In chronic skin inflammation, IL‐17 signaling drives epidermal remodeling by upregulating GLUT1 via HIF‐1α. This autonomous glycolysis in epithelial cells enhances lactate production and sustains the inflammatory loop [18]. In the context of intracerebral hemorrhage, impaired glycolysis in microglia, attributed to the downregulation of GLUT1 and hexokinase 2, promotes inflammatory responses by disrupting mitochondrial function [19].

### 2.4 Multidimensional Regulation of GLUT1 Expression and Function

Although GLUT1 constitutes the basal glucose transport machinery, its expression and activity are dynamically regulated by a complex network of signaling pathways, posttranslational modifications, and interacting proteins to adapt to physiological stresses.

#### 2.4.1 Transcriptional Control Under Stress

Cells frequently encounter physiological stressors such as low oxygen (hypoxia). The primary mediator of the adaptive response is HIF‐1α. Under hypoxic conditions, HIF‐1α serves as a key transcriptional regulator, becoming stabilized to potently drive SLC2A1 transcription and GLUT1 overexpression [24, 25, 64, 65]. This regulation is critical for metabolic adaptation, as seen in decidual cells, where SHP2 regulates GLUT1 expression through the HIF‐1α pathway [66]. The stability of HIF‐1α itself is further fine‐tuned by posttranslational modifications like lactylation, which enhances its transcriptional activity and downstream GLUT1 expression [67]. Interestingly, hypoxia can also induce the formation of a glycolytic complex involving GLUT1 and glycolytic enzymes at the plasma membrane via mechanisms independent of transcription [65].

#### 2.4.2 Signaling Pathways

The PI3K/Akt and mTOR pathways are central regulators of glucose metabolism that intimately control GLUT1. For example, in cerebral ischemia‐reperfusion injury, the knockdown of Sestrin2 (SESN2) exacerbates injury by enhancing glycolysis via the mTOR/HIF‐1α pathway, elevating GLUT1 and lactate levels [68]. This indicates that under physiological conditions, the precise regulation of the mTOR/HIF‐1α axis is crucial for maintaining metabolic homeostasis. In the kidney, pyruvate kinase M2 (PKM2) promotes the transcription of GLUT1 to regulate pericyte glycolysis during the progression of kidney disease [69]. Additionally, in the liver, ALKBH5 has been shown to regulate glucose homeostasis independently through mTORC1 signaling [13].

#### 2.4.3 Posttranslational and Membrane Dynamics

Unlike GLUT4, which relies on insulin for membrane translocation, GLUT1 is regulated by insulin‐independent mechanisms involving membrane dynamics and stability. Growth factor stimulation, such as platelet‐derived growth factor (PDGF), can regulate GLUT1‐mediated glucose uptake through endocytosis. Studies show that a portion of GLUT1 undergoes co‐endocytosis with PDGFR, trafficking to endocytic vesicles near mitochondria to potentially fuel local glycolytic machinery [70].

The stability of GLUT1 on the cell surface is strictly controlled. An increase in glucose levels can trigger the lysosomal trafficking and degradation of GLUT1 via the arrestin‐like protein TXNIP, which facilitates GLUT1 internalization [71]. Conversely, posttranslational modifications such as palmitoylation—the addition of palmitic acid to cysteine residues—are essential for regulating GLUT1 function, membrane localization, and stability [72]. Aquaporins (e.g., AQP3) can also supplement GLUT1‐mediated uptake and regulate protein modifications such as O‐GlcNAcylation to promote glycolytic flux [73].

#### 2.4.4 Novel Regulatory Partners

Emerging evidence identifies novel regulators of GLUT1. The DNA/RNA‐binding protein PURA has been found to physically form a complex with GLUT1, playing a key role in driving its function for glucose uptake [74]. In endometrial stromal cells, GLUT1 upregulation during decidualization is controlled by an epigenetic mechanism mediated by CCAAT enhancer‐binding protein β (C/EBPβ) and Wilms tumor 1 (WT1) [75]. Furthermore, the mixed‐lineage leukemia (MLL) gene regulates glucose‐sensitive gene expression, including GLUT1, in pancreatic beta cells, linking circadian rhythms to insulin secretion [76]. In chondrocytes, compounds like 5‐hydroxymethylfurfural (5‐HMF) can regulate glycolysis via the GLUT1 signaling pathway to protect against osteoarthritis [77].

**FIGURE 3:**
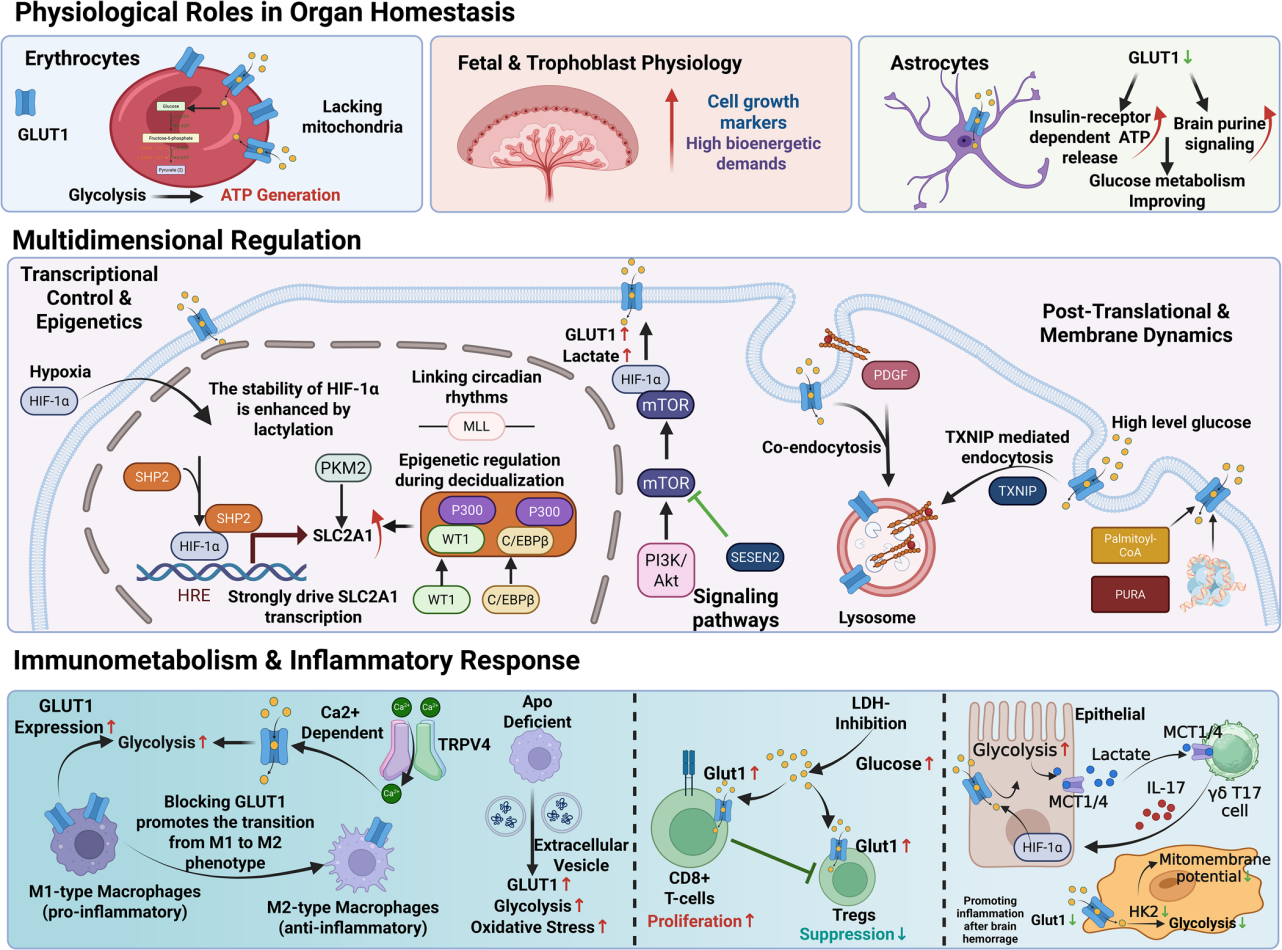
Schematic overview of GLUT1 in normal physiology: from organ homeostasis to multidimensional regulation. Beyond fueling basal mammalian physiology, such as fetal development and brain energy supply, GLUT1 plays a pivotal role in governing immunometabolism and inflammatory responses. To maintain systemic homeostasis, its expression and membrane dynamics are tightly calibrated by a sophisticated network of transcriptional controls, signaling pathways, and posttranslational modifications. *Source*: Created with bioRender.com, with permission.

Collectively, these regulatory mechanisms ensure that GLUT1 meets the metabolic demands of healthy tissues without exceeding physiological limits. The diverse physiological roles, inflammatory regulatory functions, and multidimensional regulatory networks of GLUT1 described are comprehensively summarized and depicted in Figure 3. However, when these precise control systems are disrupted by genetic mutations or chronic metabolic stress, the resulting homeostatic imbalance precipitates a spectrum of pathological conditions, as discussed in the following section.

## 3 Pathological Changes Caused by GLUT1 Dysregulation

**FIGURE 4:**
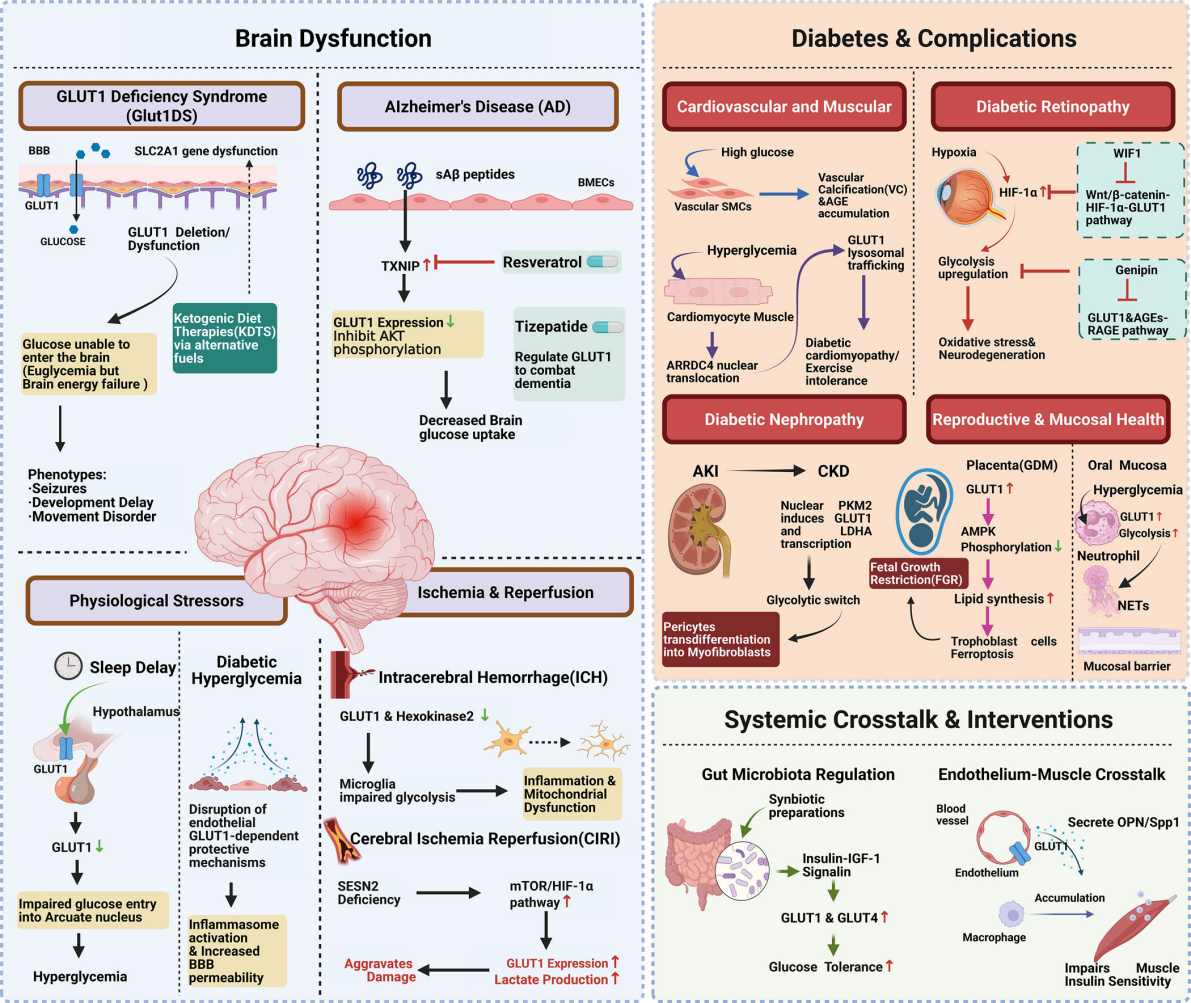
The pathogenic roles of GLUT1 dysregulation in nonmalignant diseases. Aberrant GLUT1 function disrupts systemic glucose homeostasis, triggering a wide spectrum of pathologies. These range from severe neurological energy crises in genetic Glut1 deficiency syndrome (Glut1DS) to maladaptive metabolic signaling that drives diabetic complications, neurodegenerative conditions (such as Alzheimer's disease), and ischemic injuries. *Source*: Created with bioRender.com, with permission.

Glucose transport across plasma membranes is a fundamental requisite for cellular life, and the disruption of this process precipitates a cascade of pathological events. While the solute carrier family 2 (SLC2A) genes are often discussed in the context of neoplastic transformation, the consequences of GLUT1 dysregulation extend far beyond malignancy. Defects in glucose transport are inextricably linked to a spectrum of metabolic disorders, insulin resistance, and diabetes [42, 78]. Furthermore, aberrant GLUT1 function is a critical driver of pathologies in the nervous, cardiovascular, and immune systems [19, 56, 60, 79]. The multifaceted roles of GLUT1 in maintaining the BBB integrity, modulating systemic glucose homeostasis, and driving complications in organs such as the heart, kidney, and placenta are summarized and depicted in Figure 4.

### 3.1 Genetic Deficiency: GLUT1 Deficiency Syndrome (Glut1DS)

The most indispensable physiological role of GLUT1 is found in the CNS, where it acts as the primary vehicle for glucose transport across the BBB and into astrocytes [9, 12]. The critical nature of this transport system is illustrated by Glut1DS. Caused by dysfunction in the SLC2A1 gene, this rare genetic disorder results in a “brain energy failure” characterized by a critical lack of neural fuel despite the presence of normal blood glucose levels [15, 51].

Clinically, this cerebral energy crisis manifests as a spectrum of profound neurological symptoms. Patients frequently suffer from pharmacoresistant seizures, deceleration of head growth, developmental delay, and intellectual disability [15, 51]. Additionally, complex movement disorders, including myoclonus and dysarthria, are hallmark features [15, 80, 81]. The condition may also present as stroke‐like episodes involving transient focal neurological deficits, further complicating diagnosis [82]. Because the fundamental defect lies in glucose entry, current standard treatments focus on providing alternative fuel sources for the brain, primarily through KDTs [6, 51]. While KDT remains the standard of care, research is expanding to understand the complex metabolic interactions between different brain cell types in the context of this deficiency [15].

### 3.2 Systemic Metabolic Dysregulation: Diabetes and Complications

GLUT1 exhibits complex roles in the progression of diabetes mellitus and its devastating systemic complications. At the systemic level, glucose homeostasis is governed by intricate crosstalk between tissues. For instance, insulin sensitivity is determined not only by myocytes but also by the vascular endothelium. A reduction in endothelial GLUT1 triggers the secretion of osteopontin (OPN/Spp1), which promotes macrophage accumulation and impairs muscle insulin sensitivity, highlighting a mechanism where endothelial metabolism dictates muscle glucose homeostasis [11]. Furthermore, the regulation of blood glucose involves the gut microbiota; synbiotic preparations have been shown to improve glucose tolerance by upregulating GLUT1 and GLUT4 via the insulin‐IGF‐1 signaling pathway [83].

When homeostatic control fails, hyperglycemia drives severe complications in target organs through aberrant GLUT1 signaling.

#### 3.2.1 Cardiovascular and Muscular Complications

In the cardiovascular system, vascular calcification (VC) is a critical risk factor. The peptide intermedin (IMD) has been found to alleviate high glucose‐induced calcification of vascular smooth muscle cells by inhibiting GLUT1 through the cAMP/PKA pathway, thereby reducing the accumulation of advanced glycation end products (AGEs) [84]. Similarly, in the heart and skeletal muscles, hyperglycemia promotes the nuclear translocation of ARRDC4, which blocks glucose transport by increasing lysosomal trafficking of GLUT1. Deletion of ARRDC4 restores GLUT1‐mediated transport and mitochondrial respiration, protecting against diabetic cardiomyopathy and exercise intolerance [85].

#### 3.2.2 Diabetic Retinopathy

A particularly devastating complication is diabetic retinopathy (DR), where hypoxia‐induced HIF‐1α upregulates glycolysis, causing oxidative stress and neurodegeneration. Therapeutic strategies targeting this axis show promise; for example, the overexpression of Wnt inhibitory factor 1 (WIF1) protects retinal photoreceptor cells by inhibiting the Wnt/β‐catenin‐HIF‐1α‐GLUT1 pathway [86]. Similarly, the traditional medicine component Genipin ameliorates retinal lesions by inhibiting GLUT1 and modulating the AGEs‐RAGE pathway [87].

#### 3.2.3 Diabetic Nephropathy and Kidney Disease

In the kidney, metabolic reprogramming drives the progression from acute kidney injury (AKI) to chronic kidney disease (CKD). This pathological transition involves the transdifferentiation of pericytes into myofibroblasts, a process reliant on nuclear PKM2‐induced transcription of GLUT1 and lactate dehydrogenase A (LDHA) to enable the necessary glycolytic switch [69].

#### 3.2.4 Reproductive and Mucosal Health

The pathological reach of GLUT1 dysregulation also affects reproductive health. In gestational diabetes mellitus (GDM), elevated GLUT1 expression in the placenta inhibits AMPK phosphorylation and promotes lipid synthesis, leading to ferroptosis in trophoblast cells and subsequent fetal growth restriction (FGR) [88]. Additionally, in the oral mucosa, hyperglycemia drives neutrophils to increase GLUT1 expression and glycolysis, facilitating the formation of NETs that disrupt the mucosal barrier [62].

### 3.3 Neurodegenerative and Ischemic Conditions

While GLUT1 is essential for brain homeostasis, its dysregulation is implicated in both acute injuries and chronic neurodegenerative diseases.

#### 3.3.1 Alzheimer's Disease

Alzheimer's disease (AD) is increasingly considered a metabolic disorder associated with decreased brain glucose metabolism and insulin resistance [89]. Decreased brain glucose uptake is one of the earliest signs of AD [90], and recent studies strongly support that reduced GLUT1 expression on the BBB is a primary cause of this impairment [90, 91, 92]. Clinical studies indicate that participants with AD have lower GLUT1 levels in cerebral blood vessels, which correlates with lower cognitive scores [90]. Furthermore, GLUT1 levels are negatively correlated with the levels of neuritic plaques and cerebrovascular β‐secretase‐derived fragments [90].

Mechanistically, soluble amyloid‐β (sAβ) peptides disrupt glucose transport. Exposure to sAβ peptides leads to the downregulation of GLUT1 expression in cerebral microvascular endothelial cells and inhibits AKT phosphorylation. This occurs by upregulating thioredoxin‐interacting protein (TXNIP), thereby disrupting the insulin‐AKT signaling pathway that normally regulates glucose uptake [92]. Therapeutic interventions such as resveratrol have been shown to reduce TXNIP expression and restore GLUT1 function [92]. Additionally, novel agents like Tirzepatide have shown neuroprotective effects by regulating insulin resistance markers, including GLUT1, to combat dementia in diabetic patients [93].

#### 3.3.2 Ischemia and Reperfusion Injury

Acquired defects in GLUT1 contribute significantly to acute brain injuries. In intracerebral hemorrhage (ICH), the downregulation of GLUT1 and hexokinase 2 impairs microglial glycolysis, which exacerbates the inflammatory response and mitochondrial dysfunction [19]. Conversely, in cerebral ischemia‐reperfusion injury (CIRI), the loss of SESN2 aggravates damage by excessively enhancing glycolysis via the mTOR/HIF‐1α pathway, leading to elevated GLUT1 and lactate levels [68]

#### 3.3.3 Physiological Stressors

Even systemic physiological states can impact brain GLUT1. Sleep delay has been shown to reduce GLUT1 expression in the hypothalamus, impairing glucose entry into the arcuate nucleus and leading to hyperglycemia [94]. Moreover, the integrity of the BBB is compromised in diabetes, where high glucose disrupts the GLUT1‐dependent protective mechanism in brain endothelial cells, leading to increased inflammasome activation and permeability [95].

In summary, the precise calibration of GLUT1 function is paramount to maintaining systemic health and organ homeostasis. As discussed, its dysregulation—whether manifesting as a genetic deficit in Glut1DS or as aberrant signaling in diabetic and ischemic complications—precipitates a cascade of severe neurological, metabolic, and inflammatory disorders. While these nonmalignant pathologies frequently stem from impaired, deficient, or maladaptive glucose transport, the dynamic plasticity of GLUT1 takes on an entirely different and sinister role during neoplastic transformation. Rather than suffering from a lack of transport capacity, malignant cells actively hijack and hyperactivate this machinery to fulfill their voracious metabolic demands. This stark contrast marks the transition of GLUT1 from a physiological gatekeeper to a potent oncogenic driver, the fundamental mechanisms of which are explored in the following section.

## 4 GLUT1 in Cancer: Mechanisms and Pathways

Within the oncological landscape, GLUT1 serves as a pivotal metabolic orchestrator, exerting profound influence on diverse oncogenic processes, neoplastic progression, and the remodeling of the TME. Its expression is consistently and dramatically upregulated across a multitude of human malignancies, a molecular adaptation that directly reflects the heightened glycolytic demands of rapidly proliferating tumor cells [96]. This dysregulation is not a passive consequence of transformation but a highly orchestrated process driven by the convergence of oncogenic signaling cascades and environmental pressures. Under metabolic stress such as hypoxia or oncogenic Ras activation, autophagy maintains high glycolytic activity and supports tumor proliferation by promoting GLUT1 membrane recycling [97]. The pathological upregulation of GLUT1 is predominantly mediated through HIF‐1α‐driven transcriptional activation, particularly under the hypoxic conditions that characterize solid tumor microenvironments [24]. However, this is further amplified and sustained by a sophisticated, multilayered regulatory network spanning core pro‐growth signaling pathways, epigenetic modifications, and posttranslational stability control. This intricate web of mechanisms firmly positions GLUT1 as a central node in cancer biology, where diverse oncogenic signals are translated into a potent metabolic advantage.

### 4.1 Transcriptional Control: The Convergence of Hypoxia and Oncogenic Drivers

At the transcriptional level, GLUT1 expression is governed by a cohort of powerful transcription factors that are frequently dysregulated in cancer.

The most dominant and well‐characterized regulator of GLUT1 expression is the HIF‐1α. Functioning as a master transcription factor, HIF‐1α enables cellular adaptation to low‐oxygen environments, a quintessential hallmark of solid tumors. Under normoxic conditions, HIF‐1α is continuously synthesized but rapidly targeted for proteasomal degradation. However, within the hypoxic core of the TME, its degradation is inhibited, leading to its stabilization and accumulation. Stabilized HIF‐1α translocates to the nucleus, where it dimerizes with HIF‐1β and binds to HREs located in the promoter region of its target genes, including SLC2A1, to potently drive their transcription [24, 25]. This mechanism is a conserved feature across numerous cancers, including colorectal, lung, esophageal, and pancreatic cancers, where HIF‐1α activation ensures that cells can switch to anaerobic glycolysis to survive and proliferate [98, 99, 100, 101, 102]. In colorectal cancer (CRC), for instance, hypoxia has been shown to robustly induce GLUT1 mRNA expression in both cell lines and human tissues, with GLUT1 levels directly paralleling HIF‐1α expression [103]. Similarly, Li et al. [104] elucidated a Fyn kinase‐HIF‐2α pathway mediated by the cellular prion protein (PrPc), wherein HIF‐2α—complementary to HIF‐1α—potently activates GLUT1 in CRC. In lung cancer, the HIF‐1α pathway is a critical regulator of GLUT1, and its activation contributes not only to metabolic reprogramming but also to therapeutic resistance, promoting chemoresistance in part through the upregulation of its downstream targets [105, 106]. In glioblastoma, the AMPK‐HIF‐1α signaling axis enhances glucose‐derived serine biosynthesis, further promoting tumor growth under hypoxic stress [24]. The functional importance of this pathway is further underscored by the fact that indirect therapeutic strategies targeting HIF‐1α, such as with the compound PX‐478, lead to a direct and significant reduction in GLUT1 expression and a subsequent impairment of tumor metabolism [107]. The HIF‐1α‐GLUT1 axis, therefore, represents the primary adaptive response that allows tumors to thrive in nutrient‐poor and oxygen‐deprived conditions.

Beyond the canonical hypoxia response, key oncogenic transcription factors directly amplify GLUT1 expression. The proto‐oncogene c‐Myc, a master regulator of cell growth and metabolism, has been shown to directly bind to the GLUT1 promoter to drive its expression. This is exemplified in CRC, where the condensin II subunit NCAPD3 physically interacts with both c‐Myc and E2F1 to enhance their recruitment to GLUT1 promoters, thereby driving aerobic glycolysis [108]. Similarly, in glioma, the transcription factor E2F1, regulated by TIFA, directly promotes GLUT1 expression, enhancing glycolysis and cell migration [109]. In hepatocellular carcinoma (HCC), transcription factors such as FOXM1, regulated by BTF3, directly influence GLUT1 levels, facilitating increased glucose uptake and lactate production [110, 111]. Conversely, tumor‐suppressive factors such as the Zic family protein Zic5 act as direct transcriptional repressors of SLC2A1; their loss in cancer cells leads to de‐repression of GLUT1, facilitating a glycolytic phenotype [112].

The phosphatidylinositol 3‐kinase (PI3K)/Akt/mTOR signaling cascade is a central hub for regulating cell growth, proliferation, and metabolism, and it is one of the most frequently activated pathways in human cancer. The cascade culminates in the activation of the mammalian target of rapamycin complex 1 (mTORC1), a master regulator of anabolism. mTORC1 signaling is a powerful driver of GLUT1 expression and function. It can enhance the translation of HIF‐1α mRNA, creating a potent feed‐forward loop that boosts GLUT1 transcription even under normoxia. Furthermore, mTORC1 signaling promotes the translocation of GLUT1 to the plasma membrane, increasing the cell's glucose uptake capacity [113, 114]. This critical link is evident across multiple cancers. In gastric cancer, for example, GLUT1 upregulation is tightly linked to the activation of the AKT‐mTOR axis, which promotes glycolysis and proliferation [113, 115]. In HCC, the lncRNA HOTAIR promotes glycolysis by upregulating GLUT1 via mTOR signaling, highlighting the diverse inputs into this pathway [111].

### 4.2 Posttranscriptional and Posttranslational Regulation: Fine‐Tuning GLUT1 Abundance

The control of GLUT1 is further refined at the posttranscriptional and posttranslational levels, ensuring that its expression is robustly maintained to meet the metabolic demands of the tumor.

The stability and translation of SLC2A1 mRNA are subject to exquisite epigenetic control. The N6‐methyladenosine (m6A) RNA methylation pathway plays a critical role. The m6A “writer” enzyme METTL3 has been shown to methylate SLC2A1 mRNA. This modification is then recognized by m6A “reader” proteins like IGF2BP2/3, which stabilize the transcript, shield it from decay, and enhance its translation into GLUT1 protein. The disruption of this axis via METTL3 silencing leads to reduced GLUT1 levels, suppressed glycolysis, and inhibited tumorigenesis, establishing the METTL3‐IGF2BP2/3‐GLUT1 axis as a pivotal driver of the Warburg effect [116, 117]. The abundance of functional GLUT1 protein at the cell surface is dynamically controlled by modifications that govern its stability and trafficking. The long noncoding RNA GAL (GLUT1‐associated lncRNA) physically interacts with the GLUT1 protein, promoting its SUMOylation. This modification inhibits subsequent ubiquitin‐proteasome‐mediated degradation, thereby extending the protein's half‐life and sustaining high levels of glucose transport [118].

In another layer of control, signaling pathways can modulate GLUT1 stability via autophagy. For instance, TANK‐binding kinase 1 (TBK1) has been found to promote glucose consumption by suppressing mTORC1 signaling. This mTORC1 inhibition, in turn, induces autophagy, which paradoxically decreases GLUT1 protein degradation and increases its translocation to the plasma membrane, thereby enhancing glucose uptake [119].

The regulation of GLUT1 in cancer is best understood not as the result of isolated, linear pathways but as the product of their profound synergy and intricate crosstalk. The PI3K/Akt/mTORC1 and HIF‐1α axes are deeply intertwined. As noted, mTORC1 can enhance the synthesis of HIF‐1α, creating a powerful feed‐forward loop that maximizes GLUT1 expression and locks the cell into a glycolytic state. This integration allows cancer cells to maintain a high glycolytic rate irrespective of oxygen status, providing a formidable survival advantage. This convergence is powerfully illustrated in cancers harboring specific oncogenic mutations. For instance, cancer‐associated mutations in isocitrate dehydrogenase (IDH) lead to the production of the oncometabolite 2‐hydroxyglutarate (2‐HG). 2‐HG has been shown to induce GLUT1 expression specifically through the coordinated and sequential activation of the PI3K/Akt/mTORC1‐HIF‐1α axis, demonstrating how a single genetic alteration can co‐opt this entire regulatory network [26]. This multilayered and interconnected regulatory architecture solidifies GLUT1's role as a central metabolic orchestrator, ensuring that diverse oncogenic inputs—from growth factor signaling and loss of tumor suppressors to oncometabolite production—are channeled toward a common and essential downstream effector program: the robust and sustained upregulation of glucose transport to fuel the metabolic reprogramming that is an undeniable hallmark of cancer. This central role also positions GLUT1 as a key facilitator of therapeutic resistance, as seen in pancreatic ductal adenocarcinoma (PDAC), where GLUT1 operates within a coordinated metabolic network that contributes to chemoresistance [101]. Consequently, targeting these upstream regulatory nodes or GLUT1 itself has emerged as a promising therapeutic strategy to overcome treatment resistance in refractory cancer subtypes [101].

**FIGURE 5:**
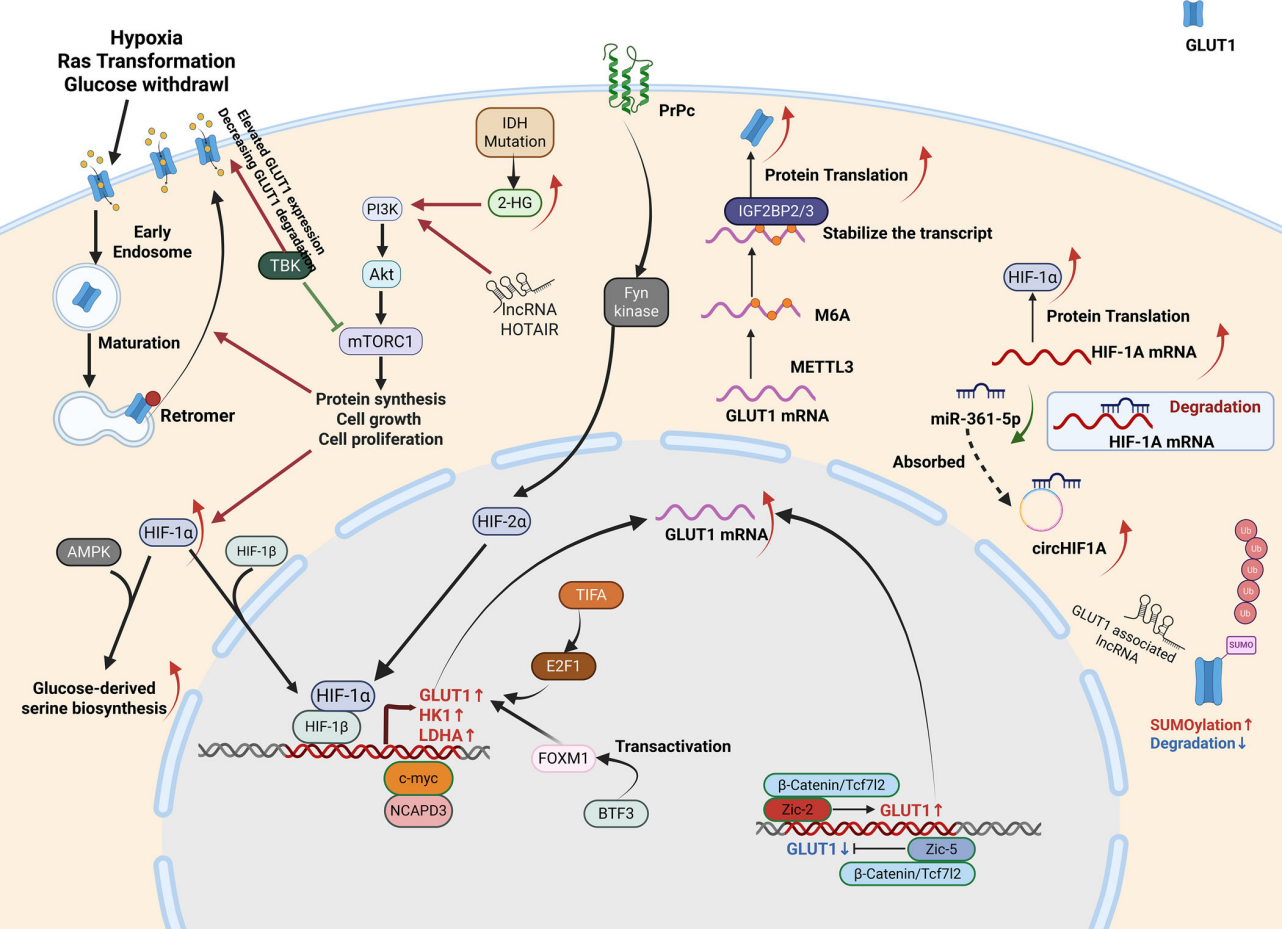
The multilayered regulatory network of GLUT1 expression in cancer. Under metabolic stress such as hypoxia, tumor cells hijack GLUT1 expression to fuel relentless aerobic glycolysis. This pathological upregulation is orchestrated by a convergence of transcriptional master regulators (e.g., HIF‐1α, c‐Myc), epigenetic modifications (e.g., m6A methylation), and posttranslational stability controls, which collectively endow the tumor with formidable metabolic plasticity. *Source*: Created with bioRender.com, with permission.

To summarize, the pathological upregulation of GLUT1 in cancer extends far beyond simple transcriptional activation; it is the culmination of a highly orchestrated, multidimensional regulatory network. As visually synthesized in Figure 5, these diverse upstream inputs—spanning transcriptional master regulators (e.g., HIF‐1α and oncogenic factors), epigenetic modifications (e.g., m6A methylation), and dynamic posttranslational control (e.g., SUMOylation and targeted degradation)—converge to lock the cancer cell into a state of relentless aerobic glycolysis. This robust regulatory architecture not only guarantees a steady supply of energy for the tumor but also endows it with formidable metabolic plasticity against environmental stress. Understanding these core biochemical mechanisms provides the essential foundation for exploring how this generalized “Warburg” machinery translates into distinct aggressive phenotypes, metastasis, and varied prognostic outcomes across different human malignancies, which we will systematically dissect in the next section.

## 5 GLUT1 Across the Oncological Spectrum: Expression, Prognosis, and Tumor‐Specific Mechanisms

**FIGURE 6:**
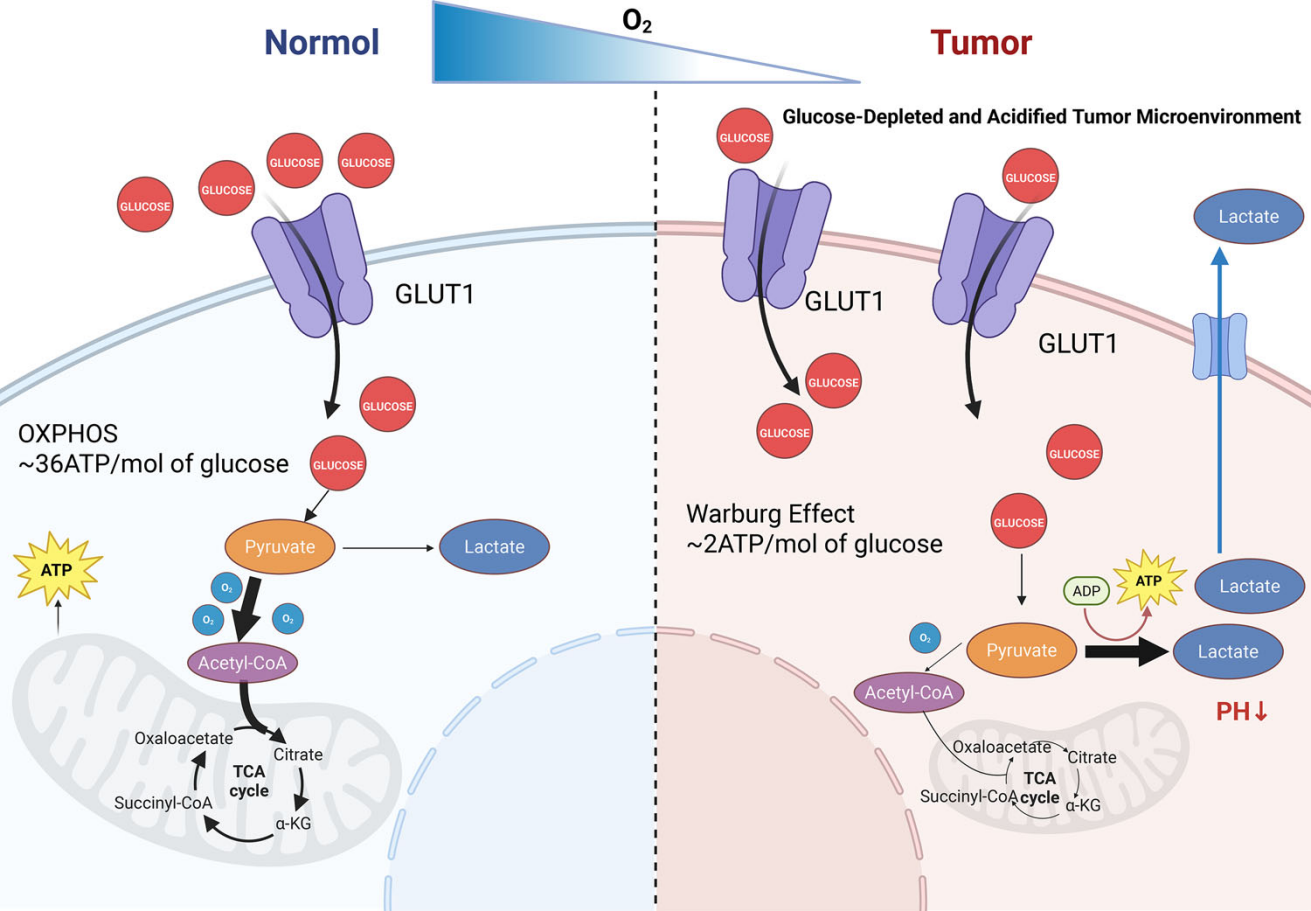
Contrasting glucose metabolism pathways between normal and malignant cells. (Left) Normal cells prioritize mitochondrial oxidative phosphorylation (TCA cycle → ATP) for energy production. (Right) Tumor cells exhibit the Warburg effect, favoring glycolytic lactate accumulation despite aerobic conditions. Key features include upregulated GLUT1 transporters and enhanced glycolytic flux. *Source*: Created with bioRender.com, with permission.

The foundational mechanisms driving GLUT1 overexpression provide a unified framework for understanding its role in cancer, yet its precise functional impact and clinical significance are profoundly shaped by the unique biological context of each malignancy. Across the oncological spectrum, from carcinomas to hematological cancers, elevated GLUT1 expression consistently emerges as a powerful prognostic biomarker, robustly correlating with aggressive tumor phenotypes, advanced disease stage, increased metastatic potential, and diminished patient survival. This widespread correlation underscores a fundamental principle: the reprogramming of cellular bioenergetics, often termed the Warburg effect, is a near‐universal hallmark of malignant progression, as depicted in Figure 6. In this metabolic landscape, tumor cells prioritize high‐rate glycolysis over mitochondrial oxidative phosphorylation, a shift that necessitates the dramatic upregulation of GLUT1 to fuel the voracious demand for glucose. Crucially, this upregulation functions not merely as a passive metabolic feature but as a dynamic dual force. It acts as a primary tumor driver, actively propelling malignant progression by fueling proliferation, metastasis, and chemoresistance while orchestrating an immunosuppressive microenvironment [56, 101, 120]. Concurrently, it serves as a vital metabolic adaptation, enabling tumor cells—and even immune effectors like engineered CAR‐T cells—to sustain survival and function amidst the nutrient deprivation or therapeutic stress characteristic of the solid tumor niche [121, 122]. While this core dependency is shared, the specific pathways that are co‐opted with GLUT1, and the resulting contributions to tumor‐specific behaviors such as invasion, chemoresistance, and immune evasion, vary significantly between cancer types. The following sections will dissect the distinct roles and regulatory nuances of GLUT1 across several major human cancers, highlighting not only its common role as a metabolic gatekeeper but also its specialized functions in driving the pathophysiology of each disease.

### 5.1 Colorectal Cancer

In colorectal cancer (CRC), an absence of GLUT1 expression is observed in normal colonic epithelium, with its emergence and intensification occurring during the adenoma–carcinoma transition [123, 124]. GLUT1 positivity increases from merely 18% in low‐grade adenomas to 63% in high‐grade adenomas, ultimately reaching near‐universal expression in invasive carcinomas [125]. This temporal pattern indicates that GLUT1 upregulation is a relatively late molecular event closely tied to malignant transformation in CRC. Clinically, elevated GLUT1 expression exhibits robust correlations with poor histological differentiation, advanced tumor staging, and an increased incidence of lymph node metastasis [125]. Patients with high GLUT1 immunostaining (>50% positivity) face a 2.4‐fold increased risk of disease‐specific mortality [123] and significantly reduced overall and disease‐free survival [124]. Furthermore, patients classified into the “Warburg‐high” subgroup—characterized by elevated expression of glycolytic proteins including GLUT1—display the worst CRC‐specific and overall survival rates, independent of conventional prognostic factors [98, 126].

The regulation of GLUT1 in CRC involves unique molecular networks. Upstream, the circular RNA circHIF1A acts as a miR‐361‐5p sponge, thereby derepressing HIF1A and upregulating GLUT1 [127]. Additionally, the pro‐proliferative protein S100A2 activates the PI3K/AKT pathway to specifically upregulate GLUT1, promoting glycolysis [128]. Downstream, GLUT1 acts as an essential mediator for the pro‐tumorigenic transcription factor HES1 to drive aerobic glycolysis and CRC progression [129]. Furthermore, GLUT1 is directly linked to tumor angiogenesis and metastasis; for instance, the lncRNA SOX2 promotes vasculogenic mimicry—the formation of vessel‐like structures by tumor cells—in a strictly GLUT1‐dependent manner [130]. Targeting GLUT1 represents a crucial therapeutic vulnerability in CRC. Direct inhibition using the GLUT1 inhibitor BAY‐876 induces a profound metabolic crisis characterized by increased mitochondrial respiration, elevated reactive oxygen species (ROS), and apoptosis, potently suppressing CRC cell growth [131]. Therapeutically, the fiber fermentation byproduct sodium butyrate has been shown to inhibit CRC growth by promoting the autophagic degradation of HIF‐1α, leading to the subsequent suppression of GLUT1 [132]. Additionally, targeting the circHIF1A‐GLUT1 axis may provide a novel strategy to overcome cetuximab resistance in CRC patients [127].

### 5.2 Breast Cancer

Breast cancer cells exhibit significant GLUT1 overexpression compared with healthy mammary epithelial cells, widely adopting the “Warburg Effect” to fuel their growth [133]. This elevated expression is generally high and particularly pronounced in aggressive subtypes, such as triple‐negative breast cancer (TNBC) [134, 135]. High GLUT1 expression robustly correlates with multiple adverse clinicopathological factors, including estrogen receptor (ER) and progesterone receptor (PR) negativity, a high Ki‐67 proliferation index, and high histological and nuclear grades [136, 137, 138]. Consequently, elevated GLUT1 is strongly associated with a poor prognosis in TNBC [134, 135] and is consistently linked to the shortening of both OS and DFS across breast cancer patients [138, 139].

Beyond fueling bioenergetic and biosynthetic demands, GLUT1 directly promotes breast cancer cell proliferation, survival, invasiveness, and metastatic ability, playing a critical role during early tumor development and metastasis [134, 140]. In TNBC, tumor growth is functionally dependent on a specific RB1‐GLUT1 metabolic axis [135]. Furthermore, emerging evidence highlights a unique connection between IL‐4 receptor signal transduction and GLUT1, which drives breast cancer metabolic modulation [139]. The RB1‐GLUT1 metabolic axis represents a key therapeutic vulnerability; specifically, GLUT1 inhibition potently blocks the growth of RB1‐positive TNBC cells. This highlights RB1 expression as a potential predictive biomarker to stratify patients for personalized GLUT1‐targeted therapies [134, 135]. Additionally, disrupting interconnected networks, such as the IL‐4 receptor signaling pathway, offers promising novel strategies for targeting breast cancer metabolism [139].

### 5.3 Lung Cancer

GLUT1 is frequently overexpressed in NSCLC, including both lung adenocarcinoma (LUAD) and lung squamous cell carcinoma (LUSC) [141, 142]. Notably, its expression exhibits distinct nuances between these subtypes: in LUAD, GLUT1 overexpression is closely tied to intrinsic tumor progression, whereas in LUSC, it shows a stronger correlation with specific patterns of immune cell infiltration, including B cells, CD8+ T cells, CD4+ T cells, and macrophages [142]. Elevated GLUT1 serves as a key indicator of tumor aggressiveness and is consistently associated with poor survival rates in patients with advanced lung cancer [141, 142]. Specifically, in LUSC patients receiving immunotherapy, high GLUT1 expression correlates with a poor pathological response and shortened survival [105, 141].

Under hypoxic conditions, increased GLUT1 expression drives profound metabolic alterations that shape an immunosuppressive TME [143, 144]. This enhanced intracellular glucose metabolism reprograms the TME, affecting the infiltration and function of tumor‐infiltrating lymphocytes (TILs) and thereby contributing to immune evasion [142]. Furthermore, GLUT1 functionally mitigates oxidative stress, a mechanism directly linked to the development of chemoresistance in lung cancer cells [102]. Given its role in metabolic reprogramming and drug resistance, targeting GLUT1 is a viable strategy to improve treatment efficacy [143, 145]. GLUT1 remains significantly upregulated in cisplatin‐resistant lung cancer cell lines, and its inhibition enhances the apoptotic response to cisplatin [102]. For example, the agent xanthatin effectively reduces GLUT1 expression, which enhances the accumulation of reactive oxygen species (ROS) and successfully induces apoptosis in cisplatin‐resistant cells [102].

### 5.4 Esophageal Cancer

In esophageal cancer, particularly esophageal squamous cell carcinoma (ESCC), GLUT1 exhibits high expression that strongly correlates with aggressive tumor characteristics, including increased depth of invasion, a higher incidence of lymph node metastasis, and advanced TNM staging [146, 147, 148]. GLUT1 positivity serves as an independent prognostic biomarker for increased risk of relapse and lower overall survival rates [100, 146]. Patients with high GLUT1 expression consistently exhibit higher rates of recurrence, significantly shorter survival times, and an elevated relative risk of death compared with those with lower expression levels [100, 147, 149].

Unique to ESCC, GLUT1 is essential for maintaining cancer stem cell‐like properties by inhibiting autophagy‐dependent ferroptosis. It achieves this by directly interacting with and stabilizing the epidermal growth factor receptor (EGFR), which promotes pro‐survival pathways [150]. Additionally, GLUT1‐driven metabolic activity correlates with key angiogenesis markers like VEGF, supporting increased microvessel density [151]. This enhanced angiogenesis facilitates tumor growth and is strongly associated with a higher incidence of hematogenous recurrence [149, 151]. High GLUT1 expression confers a survival advantage that contributes to chemoresistance in ESCC. Functionally, GLUT1 inhibition reduces cell proliferation and critically increases the efficacy of chemotherapy, especially cisplatin [100]. Clinically underscoring its predictive value, patients with GLUT1‐negative tumors demonstrate a significantly greater reduction in standardized uptake values (SUV) on posttreatment PET imaging, indicating a vastly improved metabolic response to therapy [100].

### 5.5 Gastric Cancer

In gastric cancer, a significant increase in GLUT1 expression is closely associated with tumor progression and malignancy. Clinically, elevated GLUT1 levels are significantly associated with increased tumor invasiveness, a higher incidence of lymph node metastasis, and poor tumor differentiation [152, 153]. Notably, this high expression extends to specific histological subtypes, being particularly correlated with mucinous adenocarcinoma [154, 155]. A large body of evidence and multiple meta‐analyses support GLUT1 as a robust poor prognostic indicator for gastric cancer. High expression is significantly correlated with reduced OS and DFS [152, 155, 156]. Critically, elevated GLUT1 has been identified as an independent factor for poor prognosis, highlighting its importance in clinical risk stratification [114, 155].

High levels of GLUT1 provide the necessary energy to support the rapid proliferation, migration, and invasion of gastric tumor cells [114, 115]. While canonical pathways like the PI3K/Akt/mTOR and HIF‐1α axes are active drivers [114, 115], GLUT1 regulation in this malignancy is uniquely distinguished by microRNA (miRNA) networks. In a complex interaction, miR‐520a‐3p indirectly affects GLUT1 function by targeting AKT1, a key upstream kinase in the PI3K/Akt/mTOR pathway [115], intimately intertwining GLUT1 expression with broader noncoding RNA signaling. The reliance of gastric cancer on GLUT1‐driven energy metabolism presents a distinct metabolic vulnerability. Specifically, manipulating the aforementioned miRNA regulatory networks can effectively disrupt the glycolytic process; for example, the upregulation of miR‐148b can directly inhibit the expression of GLUT1, consequently reducing the glycolytic capacity of cancer cells [113].

### 5.6 Prostate Cancer

Elevated GLUT1 expression characterizes advanced prostate cancer stages, correlating significantly with poor differentiation (higher Gleason scores) and advanced pTNM staging [157, 158]. Furthermore, GLUT1 levels are positively correlated with the cellular proliferation marker Ki‐67 [159, 160]. Clinically, elevated GLUT1 levels (over 19.1% positivity) serve as an independent negative prognostic marker for biochemical recurrence postradical prostatectomy [161]. Its strong association with other pathological factors, such as high serum prostate‐specific antigen (PSA) levels, further cements its role as a robust marker for aggressive disease, correlating with poor overall survival and a greater likelihood of recurrence [161].

Functionally, GLUT1 facilitates metabolic reprogramming within the hypoxic tumor microenvironment [162, 163] and mediates cell cycle progression, as evidenced by reduced proliferation following GLUT1 knockdown [160]. A distinctly unique feature of GLUT1 regulation in prostate cancer is its modulation by androgens; androgen‐responsive cells (e.g., LNCaP) exhibit distinct GLUT1 upregulation, intertwining hormonal signaling with metabolic rewiring [164]. Furthermore, GLUT1 plays a vital protective role by shielding prostate cancer cells from glucose deprivation‐induced oxidative damage in nutrient‐poor conditions [162]. Targeting this metabolic transporter could potentially improve clinical outcomes in patients with aggressive prostate cancer [161]. The unique androgen‐GLUT1 connection presents specific therapeutic opportunities aimed at inhibiting glucose metabolism in androgen‐sensitive tumors [164]. Additionally, impeding GLUT1 function decreases tumor growth and sensitizes cancer cells to concurrent treatments. For example, inhibiting EGFR signaling effectively reduces GLUT1 levels, thereby suppressing tumor cell proliferation under glucose‐deprived conditions and highlighting the therapeutic vulnerability of interconnected oncogenic pathways [165].

### 5.7 Bladder Cancers

The upregulation of GLUT1 is a critical adaptation for bladder cancer cells to meet their high energetic demands via glycolysis [166]. Its elevated expression is frequently observed in conjunction with the upregulation of HIF‐1α within the hypoxic tumor microenvironment, robustly correlating with higher tumor grades and advanced stages [167]. High GLUT1 expression serves as a significant prognostic biomarker, particularly in patients with invasive bladder cancer. Clinically, elevated GLUT1 levels are strongly indicative of aggressive tumor characteristics and are associated with significantly poorer overall survival and recurrence‐free survival [167].

Beyond the canonical HIF‐1α pathway, bladder cancer metabolic reprogramming is uniquely driven by the SIRT1/GLUT1 axis, which promotes cell proliferation and a glycolytic phenotype [166]. Uniquely, GLUT1‐facilitated glucose uptake promotes the epithelial–mesenchymal transition (EMT), involving the upregulation of oncogenic factors YAP1 and TAZ. These factors, in turn, further amplify GLUT1 expression, creating a vicious feedback loop that directly links metabolism with invasiveness and metastasis [168]. Additionally, GLUT1 influences glycogen metabolism in bladder cancer cells, where tumor‐promoting enzymes such as glycogen phosphorylase are dysregulated alongside GLUT1 to fuel aberrant energy production [169]. Inhibition of GLUT1 activity using small‐molecule inhibitors has demonstrated the potential to directly restrict bladder tumor growth and metastasis, suggesting new therapeutic avenues [170]. Furthermore, exploiting endogenous regulatory networks presents a viable intervention strategy; for instance, the direct transcriptional repression of GLUT1 by microRNAs, such as miR‐340, successfully reduces cell proliferation and increases apoptosis, offering a molecular mechanism that could be targeted to improve clinicopathological outcomes [171].

### 5.8 Liver Cancers

In hepatocellular carcinoma (HCC), the most common form of liver cancer, GLUT1 is frequently overexpressed compared with normal liver tissues, correlating strongly with enhanced glycolytic activity [110]. Clinically, elevated GLUT1 levels are predominantly enriched in poorly differentiated tumors rather than well‐differentiated ones [172]. High GLUT1 expression correlates significantly with advanced tumor stages and the presence of metastasis, positioning it as a robust biomarker for disease progression [173, 174]. Consequently, patients with elevated GLUT1 levels exhibit significantly poorer overall survival and shorter time to recurrence [172]. Notably, alongside other metabolic indicators such as MCT4, GLUT1 serves as a highly valuable prognostic biomarker for predicting tumor aggressiveness and patient outcomes [172].

A uniquely identifying feature of HCC metabolic reprogramming is the regulation of GLUT1 by the enzyme dihydrolipoyl transacetylase (DLAT), which mechanistically drives the epithelial‐to‐mesenchymal transition (EMT) and the metastatic cascade [175]. Furthermore, GLUT1‐driven glucose consumption significantly shapes the TME. By altering local glucose availability and fostering hypoxia, HCC cells alter the phenotype of surrounding stromal cells to create an immunosuppressive niche [79, 176]. Because GLUT1 orchestrates a hypoxic and immunosuppressive microenvironment that actively shields the tumor from immune attack [79, 176], targeting GLUT1‐mediated metabolism presents a strategic therapeutic vulnerability. Disrupting this metabolic node offers a potential avenue to not only halt the DLAT‐driven metastatic cascade [175] but also reverse immune evasion in HCC.

### 5.9 Cervical Cancers

In cervical cancer, particularly in cases associated with high‐risk human papillomavirus (HR‐HPV) infections, GLUT1 is notably upregulated. Serving as a dynamic biomarker of disease progression, GLUT1 levels significantly increase as lesions progress from low‐grade cervical intraepithelial neoplasia (CIN) to high‐grade lesions and ultimately to invasive cervical carcinoma (ICC) [177]. Additionally, higher GLUT1 levels are consistently associated with larger tumor sizes and more advanced FIGO stages [178]. Elevated GLUT1 expression is a significant prognostic marker for poor overall survival (OS) in both HPV‐positive and HPV‐negative populations, though the adverse effects are often more pronounced in HPV16‐positive cohorts [179]. Specifically, tumors exhibiting high GLUT1 are more likely to metastasize [180], and their elevation is directly linked to reduced progression‐free survival (PFS) in patients undergoing primary radiation therapy [99]. Conversely, low or absent GLUT1 expression indicates a more favorable prognosis and longer metastasis‐free survival [180].

A uniquely defining feature of cervical cancer is that its adaptive metabolic response—characterized by the co‐expression of GLUT1 with other metabolic proteins such as LDHA and monocarboxylate transporter‐4 (MCT4)—is significantly influenced by HR‐HPV infection [177]. Furthermore, GLUT1 directly shapes an immunosuppressive microenvironment; its high expression is associated with decreased infiltration of critical immune cells, such as CD8+ T cells and B cells, creating an immune‐privileged niche that facilitates tumor progression and evasion from immune surveillance [179]. GLUT1 is deeply implicated in the mechanisms of therapeutic resistance in cervical cancer. Notably, the co‐expression of GLUT1 with CD147 is strongly correlated with radiation resistance, modifying tumor behavior and maintaining a high glycolytic flux under the stress of radiotherapy [99, 177]. This dependency highlights GLUT1 as a critical strategic target for overcoming radioresistance in therapeutic interventions [180].

### 5.10 Pancreatic Ductal Adenocarcinoma and Other Solid Tumors

In solid tumors such as pancreatic ductal adenocarcinoma (PDAC) and oral squamous cell carcinoma (OSCC), high GLUT1 expression is a core feature of disease progression. Elevated GLUT1 levels correlate with advanced tumor stages in PDAC [181, 182] and are closely linked to tumor invasion and larger tumor sizes in OSCC [183]. Consistently, high GLUT1 levels serve as a strong indicator of poor patient outcomes across these malignancies, including PDAC [181, 182] and glioma [184]. Furthermore, in OSCC, increased GLUT1 expression is specifically associated with aggressive cellular proliferation and therapy resistance [183].

While hypoxia‐induced HIF‐1α universally upregulates GLUT1 to facilitate glycolysis in PDAC [181], glioma [185], and OSCC [183, 186], each tumor exhibits unique regulatory features. Uniquely in PDAC, a distinct circular RNA, circZNF609, upregulates GLUT1 via the miR‐378h pathway to enhance cell viability and invasion [187]. Additionally, GLUT1‐mediated glycolysis in PDAC‐associated macrophages directly correlates with tumor immunosuppression [186]. In OSCC, the transcriptional repression of miR‐340 raises GLUT1 expression to fuel rapid proliferation [188], while oncogenic transcription factors such as HIF‐1α establish a feedback loop that promotes survival under metabolic stress [183, 186]. The reliance on GLUT1 presents actionable therapeutic vulnerabilities, particularly in overcoming treatment resistance. For example, in drug‐resistant OSCC cells, the combination of GLUT1 blockade with cisplatin treatment has shown significant promise in sensitizing these cells to chemotherapy, highlighting GLUT1's functional role as a target to reverse drug resistance [183].

### 5.11 Hematological Malignancies

In hematological malignancies, particularly multiple myeloma and B‐cell acute lymphoblastic leukemia (B‐ALL), the critical upregulation of GLUT1 is fundamentally linked to the metabolic reprogramming required to maintain a high glycolytic state in these liquid tumors [189, 190, 191, 192]. GLUT1 expression levels are robust indicators of patient prognosis in hematological cancers. In multiple myeloma, high expression of GLUT1 is significantly associated with shorter progression‐free survival (PFS) [193]. Similarly, the critical dependency of B‐ALL on GLUT1 highlights its profound importance in determining tumor prognosis [194].

In multiple myeloma, the targeted upregulation of GLUT1 directly promotes the survival and proliferation of myeloma cells [189, 190]. In B‐ALL, leukemic cells strictly rely on GLUT1 to maintain their high glycolytic baseline; consequently, a deficiency in GLUT1 forces an unfavorable metabolic reprogramming that intrinsically inhibits their growth and promotes apoptosis [191, 195]. Targeting GLUT1 presents a promising strategy to overcome specific chemoresistance. In multiple myeloma, GLUT1 enhances resistance to chemotherapy drugs such as melphalan and platinum phosphate [189, 190]; inhibiting GLUT1 activity induces apoptosis and significantly enhances the efficacy of these chemotherapies [189]. In B‐ALL, utilizing metabolic inhibitors such as 2‐deoxyglucose or implementing experimental GLUT1 blockade successfully reduces cellular proliferation, increases apoptosis rates, and slows tumor progression [191, 192, 194].

These diverse, cancer‐specific roles, prognostic implications, and regulatory mechanisms are consolidated in Table 1 for a comparative overview.

**TABLE 1:** GLUT1 across the oncological spectrum: A comparative overview.

Cancer type	GLUT1 Expression pattern	Prognostic significance	Key mechanistic roles	Notable regulatory features	Relevant references
Colorectal cancer (CRC)	Progressively increases with malignancy	High expression indicates poor prognosis, advanced stage, and reduced survival	Fuels proliferation (Warburg effect); promotes metastasis and vasculogenic mimicry	HIF‐1α axis, PI3K/AKT pathway, modulated by noncoding RNAs and oncogenic proteins (S100A2)	[98, 123, 124, 125, 126, 127, 128, 129, 130, 131, 132]
Breast cancer	High, especially in aggressive subtypes (e.g., TNBC)	A robust marker for poor outcomes and shortened overall and disease‐free survival	Drives proliferation, invasion, and metastasis; represents a key therapeutic vulnerability	Regulated by the RB1‐GLUT1 metabolic axis and IL‐4 receptor signaling	[133, 134, 135, 136, 137, 138, 139, 140]
Lung cancer	Frequently overexpressed; patterns differ by subtype	Correlates with poor survival rates and poor response to immunotherapy	Promotes resistance to chemotherapy (cisplatin) and radiotherapy; shapes an immunosuppressive microenvironment	Expression in LUSC is strongly linked to immune cell infiltration patterns	[102, 105, 141, 142, 143, 144, 145]
Esophageal squamous cell carcinoma	High expression correlates with aggressive tumor features	An independent predictor of poor clinical outcomes, higher recurrence, and shorter survival	Confers chemoresistance; maintains cancer stem cell properties; supports angiogenesis	Interacts with and stabilizes EGFR, promoting pro‐survival pathways	[100, 146, 147, 148, 149, 150, 151]
Gastric cancer	Significantly increased expression	An independent factor for poor prognosis, correlating with invasion and metastasis	Fuels rapid tumor cell proliferation, migration, and invasion	Driven by PI3K/Akt/mTOR and HIF‐1α axes; distinctly regulated by microRNAs (e.g., miR‐148b)	[113, 114, 115, 152, 153, 154, 155, 156]
Prostate cancer	Elevated in advanced disease stages	An independent negative marker for biochemical recurrence and poor overall survival	Mediates cell proliferation; protects cells from oxidative stress induced by glucose deprivation	A distinguishing feature is its modulation by androgens	[157, 158, 159, 160, 161, 162, 163, 164, 165]
Bladder cancer	Upregulated, correlating with high tumor grade and stage	A reliable biomarker for tumor aggressiveness and poor patient outcomes	Drives the epithelial–mesenchymal transition (EMT), enhancing metastatic potential	Regulated by HIF‐1α and SIRT1 axes; involved in a feedback loop with YAP1/TAZ	[167, 168, 169, 170, 171]
Liver cancers	Frequently overexpressed, especially in poorly differentiated tumors	Correlates with aggressive tumor behavior, metastasis, and significantly poorer survival	Drives EMT and metastasis; helps shape a hypoxic, immunosuppressive microenvironment	Regulated by the enzyme dihydrolipoyl transacetylase (DLAT)	[79, 172, 173, 174, 175, 176]
Cervical cancers	Upregulated, increasing with lesion progression (especially in HR‐HPV+ cases)	A dynamic biomarker for disease progression and a predictor of poor overall survival	Implicated in radiation resistance; contributes to an immunosuppressive microenvironment	Co‐expression with CD147 is linked to radiation resistance; influenced by HR‐HPV infection	[99, 177, 178, 179, 180]
PDAC and other solids	Commonly overexpressed in PDAC, Glioma, and OSCC	Correlates with advanced stages and poor patient outcomes	Supports nutrient uptake, invasion, proliferation, and therapy resistance	Primarily regulated by HIF‐1α in response to tumor hypoxia	[181, 182, 183, 184, 185, 186, 187, 188]
Hematological	Upregulated in multiple myeloma and B‐ALL	In multiple myeloma, high expression is linked to shorter progression‐free survival	Promotes survival, proliferation, and chemoresistance; essential for the glycolytic state of leukemia cells	Not specified in the provided text	[189, 190, 191, 192, 193, 194, 195]

## 6 GLUT1 in Tumor Microenvironment Remodeling

**FIGURE 7:**
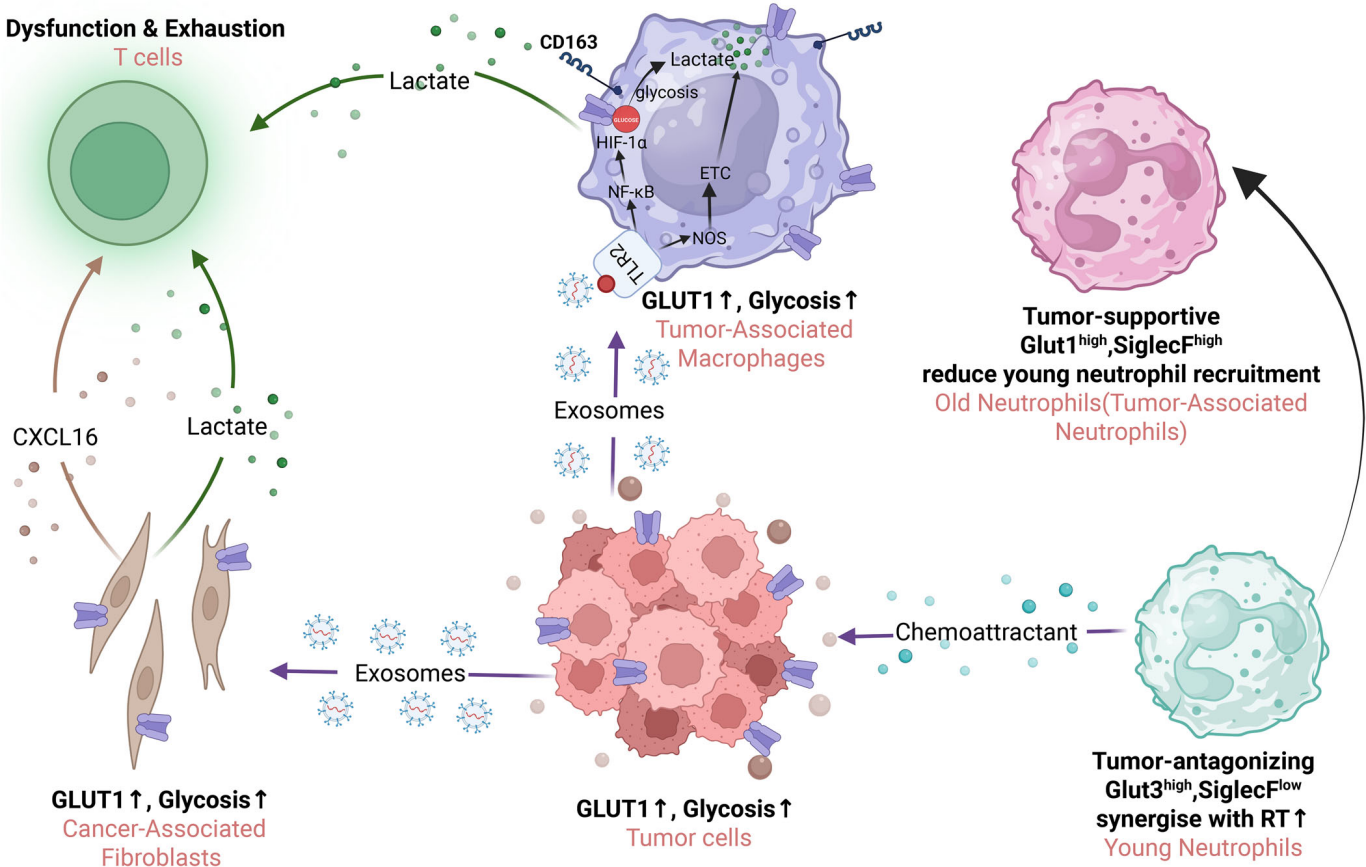
Remodeling of the tumor microenvironment (TME) by GLUT1. GLUT1 overexpression in tumor cells promotes a glycolytic phenotype, leading to the secretion of metabolites like lactate. This reshapes the TME by inducing T‐cell dysfunction, reprogramming macrophages and cancer‐associated fibroblasts (CAFs), and influencing neutrophil dynamics, ultimately fostering an immunosuppressive and pro‐tumorigenic niche. *Source*: Created with bioRender.com, with permission.

The influence of GLUT1 extends beyond the cancer cell itself, profoundly reshaping the TME, a complex milieu of stromal, immune, and endothelial cells. This remodeling is driven by a fundamental metabolic shift: the intense glucose consumption by both GLUT1‐overexpressing cancer cells and distinct populations of tumor‐resident immune cells. Notably, pro‐tumorigenic SPP1+ macrophages exhibit high preferential expression of GLUT1, a state facilitated by the HIF‐1α pathway, which promotes their polarization toward an immunosuppressive M2 phenotype [141, 196]. This combined glucose competition creates a glucose‐depleted, or “hypoglycemic,” and lactate‐rich acidic TME [61, 196]. This altered metabolic landscape, in turn, triggers the reprogramming of other key stromal cells. For instance, the interaction of CAFs with tumor‐derived exosomes induces metabolic and phenotypic changes, activating them to further promote tumor progression, angiogenesis, and remodeling of the extracellular matrix [197]. This complex interplay, illustrated in Figure 7, highlights how GLUT1's influence extends beyond the cancer cell to orchestrate the entire tumor ecosystem.

### 6.1 Cancer‐Associated Fibroblasts

As central players in this remodeled TME, CAFs engage in metabolic crosstalk with cancer cells, enhancing tumor aggressiveness. In lung cancer, reciprocal interactions lead to the upregulation of GLUT1 in CAFs, which increases their glucose uptake and glycolytic activity, allowing them to release lactate and other metabolites that fuel adjacent tumor cells [198]. Furthermore, elevated GLUT1 expression contributes to an immunosuppressive microenvironment, as glycolytic CAFs (glyCAFs) can impede cytotoxic T‐cell infiltration through mechanisms involving GLUT1‐dependent CXCL16 expression [199]. In liver and colorectal cancer, GLUT1 is implicated in metastasis, where the activation of hepatic stellate cells (HSCs) into myofibroblast‐like CAFs correlates with increased GLUT1 expression and accelerated tumor progression [33, 200]. Consequently, elevated GLUT1 levels in CAFs are associated with increased metastatic potential and poor clinical outcomes [201].

This profound metabolic dependency presents a key therapeutic vulnerability. Inhibiting GLUT1 function can disrupt the metabolic support provided by CAFs, thereby sensitizing tumors to therapy. Recent strategies have explored the use of CAF‐derived extracellular vesicles loaded with GLUT1 inhibitors to remodel the TME and reinvigorate antitumor immune responses [202, 203]. Integrating GLUT1 inhibitors with conventional chemotherapies or immunotherapies may produce synergistic effects, enhancing therapeutic responses in tumors that are otherwise resistant due to metabolic support from CAFs [204, 205]. A deeper understanding of the specific roles of GLUT1 in different tumor contexts will be critical for developing personalized treatment strategies tailored to the metabolic profile of individual tumors [206, 207].

### 6.2 Tumor‐Associated Neutrophils

Tumor‐associated neutrophils (TANs) can exhibit both pro‐ and antitumor functions depending on signals from the local microenvironment. TANs‐expressing GLUT1 demonstrate enhanced glucose metabolism, which is critical for their tumor‐supportive behavior. In a study on lung adenocarcinoma, the downregulation or loss of GLUT1 in TANs resulted in accelerated neutrophil turnover and reduced tumor growth, coupled with increased radiotherapy efficacy [143]. This suggests that GLUT1 acts as a metabolic checkpoint for TAN function, influencing their ability to support tumor growth. The mechanism by which GLUT1 enhances TAN functionality includes facilitating increased glucose uptake, which is pivotal for their metabolic reprogramming. Enhanced glycolytic activity allows TANs to alter their behaviors in the tumor microenvironment, potentially increasing their pro‐tumor functions. For instance, under hyperglycemic conditions, neutrophils are more likely to expel NETs, further exacerbating local inflammation and potentially aiding tumor immunity [62]. Future clinical applications should focus on quantifying GLUT1 expression in TANs and utilizing biomarkers that reflect metabolic reprogramming within tumors. Such strategies could inform treatment decisions, tailoring therapies based on tumor metabolism and immune cell profiles [27, 143].

### 6.3 Tumor‐Associated Macrophages

Tumor‐associated macrophages are polarized into different phenotypes, broadly classified into pro‐inflammatory M1 and anti‐inflammatory M2 subtypes. The M2 phenotype is often encouraged by key cytokines such as IL‐4 and IL‐10, promoting tissue repair and immune suppression, which is beneficial for tumor growth [208, 209]. Evidence shows that despite their M2 polarization, TAMs maintain high glycolytic activity, similar to M1 macrophages, which is driven by GLUT1 expression. This metabolic adaptation allows them to produce lactate, further contributing to the immunosuppressive environment by inducing T‐cell dysfunction [60, 208]. High GLUT1 levels in TAMs have been associated with poor prognosis, indicating their role in fostering a supportive microenvironment for tumor growth. For instance, in muscular‐invasive urothelial carcinoma (MIUC), a high density of CD163+ macrophages (indicative of M2 TAMs) was linked to increased GLUT1 expression, correlating with adverse patient outcomes [168].

### 6.4 Harnessing GLUT1 Expression to Enhance CAR‐T Cell Therapy

Recognizing this metabolic hurdle, a novel therapeutic strategy involves genetically engineering CAR‐T cells to overexpress GLUT1, thereby “arming” them for survival and function in the hostile, glucose‐poor TME. Enforced expression of GLUT1 in CAR‐T cells provides them with a competitive advantage, leading to enhanced metabolic fitness, resistance to exhaustion and apoptosis, and improved antitumor efficacy. GLUT1‐overexpressing CAR‐T cells demonstrate increased glycolytic capacity and enhanced mitochondrial oxidative phosphorylation, suggesting a broad improvement in their ability to utilize glucose for energy and biosynthesis [122, 196]. This metabolic enhancement translates directly to superior tumor control. In preclinical models of acute lymphoblastic leukemia (ALL), renal cell carcinoma (RCC), glioblastoma (GBM), and hepatocellular carcinoma (HCC), GLUT1‐overexpressing CAR‐T cells showed significantly improved tumor clearance, prolonged survival, and better responses to tumor rechallenge compared to conventional CAR‐T cells [196, 210]. By ensuring adequate energy supply, GLUT1 overexpression helps protect CAR‐T cells from activation‐induced cell death and diminishes transcriptional signatures associated with T‐cell exhaustion [196, 210]. Beyond immediate cytotoxicity, GLUT1 overexpression promotes the formation of T stem cell‐like memory (Tscm) phenotypes. These cells are associated with long‐term persistence and durable antitumor responses. This effect is linked to the upregulation of key memory‐associated genes such as KLF2, CCR7, and SELL [122, 196].

Collectively, the evidence reviewed in this section underscores that GLUT1 overexpression orchestrates a critical mechanism of “metabolic competition” that fundamentally reshapes the TME landscape. By aggressively monopolizing glucose uptake, GLUT1‐high tumor cells enforce a dual‐stress environment characterized by severe hypoglycemia and lactate accumulation. This metabolic disparity disproportionately impairs the function of antitumor effector T cells, which rely heavily on glycolysis for cytotoxicity, while paradoxically favoring the expansion and activation of immunosuppressive populations—including Tregs, M2‐polarized TAMs, and CAFs—that are metabolically rewired to utilize lactate or thrive under nutrient deprivation. Consequently, GLUT1 acts as the architect of a self‐reinforcing metabolic feedback loop that not only sustains tumor bioenergetics but actively fortifies an immune‐privileged niche, thereby driving therapeutic resistance and immune evasion.

## 7 Antitumor Effects of GLUT1 Inhibition

Inhibition of GLUT1 effectively curtails glucose uptake in malignant cells, a metabolic disruption that not only induces apoptosis but also significantly sensitizes tumors to conventional therapies. Mechanistically, this blockade induces profound cellular stress, manifesting as severe bioenergetic stress, perturbed mitochondrial structure, altered membrane dynamics, and the accumulation of autophagosomes, ultimately culminating in apoptosis [211]. Furthermore, this metabolic rewiring toward oxidative phosphorylation triggers the accumulation of reactive oxygen species (ROS). For example, agents like xanthatin reduce GLUT1 expression and promote ROS accumulation, thereby inducing apoptosis in cisplatin‐resistant lung cancer cells [102]. Crucially, this ROS buildup directly enhances tumor cell vulnerability to immune attack and potentiates cell death mediated by tumor necrosis factor‐alpha (TNF‐α) from cytotoxic T cells. Consequently, the genetic or pharmacological inactivation of GLUT1 not only exerts a direct cytotoxic effect but also sensitizes tumors to antitumor immunity, synergizing powerfully with anti‐PD‐1 immunotherapy [120]. GLUT1 inhibition also profoundly remodels the TME by reducing glucose consumption in tumor cells, thereby redistributing this vital nutrient to tumor‐infiltrating immune cells and enhancing antitumor immunity—a principle similarly supported by observations of LDH inhibition [61]. Additionally, GLUT1 blockade can induce G2/M cell‐cycle arrest and activate autophagy, as seen in thyroid cancer models, highlighting the complex interplay between metabolic disruption and cellular death programs [212].

### 7.1 Small‐Molecule Inhibitors

The central role of GLUT1 in tumor metabolism has established it as a compelling therapeutic target. Current pharmacological strategies primarily utilize small‐molecule inhibitors to disrupt its function via direct or indirect mechanisms.

#### 7.1.1 Direct Inhibitors

Direct inhibitors are classified based on their origin and mode of action. Noncompetitive inhibitors, such as naturally occurring methylxanthines (caffeine and theophylline), bind to an exofacial regulatory site to allosterically alter the kinetics of glucose transport, leading to inhibited uptake without preventing glucose binding [213]. Natural products, including the flavonoids phloretin and luteolin, also inhibit GLUT1‐mediated glucose uptake across various tissues [214]. Furthermore, a growing number of synthetic small molecules offer potent and selective GLUT1 blockade. WZB117 selectively limits glucose metabolism by impairing transport, which downregulates key glycolytic proteins to overcome drug resistance in imatinib‐resistant gastrointestinal stromal tumors [215], resensitizes radioresistant breast cancer cells to radiation [216], induces necrosis in neuroblastoma, and acts synergistically with the tyrosine kinase inhibitor apatinib in melanoma models [217, 218]. BAY‐876 is another highly selective inhibitor that strongly disrupts cancer cell bioenergetics; it is particularly effective against highly glycolytic RB1‐positive triple‐negative breast cancer (TNBC) cells [135], and its efficacy can be further enhanced in combination with bitter taste receptor agonists in head and neck squamous cell carcinoma [219]. Other direct inhibitors include KL11743 and STF‐31; notably, STF‐31 exhibits a dual mechanism of action by suppressing glucose uptake in highly dependent tumor cells and concurrently inhibiting nicotinamide phosphoribosyltransferase (NAMPT) in NAD salvage pathways [189, 220, 221].

#### 7.1.2 Indirect Suppression

An alternative strategy involves targeting upstream transcriptional regulators of GLUT1. The compound PX‐478, for example, inhibits hypoxia‐inducible factor 1α (HIF‐1α) in hypoxic conditions. By reducing HIF‐1α activity, PX‐478 administration leads to a corresponding decrease in GLUT1 expression and impairs downstream metabolic processes, including ATP production [107].

#### 7.1.3 Overcoming Resistance and Combination Therapies

Therapeutic resistance to GLUT1 inhibition remains a significant challenge, often arising from the compensatory upregulation of other glucose transporters, such as GLUT2 or GLUT4 [222]. To overcome this metabolic plasticity, GLUT1 inhibitors are rationally combined with conventional chemotherapy. For instance, GLUT1 blockade enhances the efficacy of cisplatin in head and neck cancer models [223]. Similarly, combining STF‐31 with cisplatin produces a marked synergistic cytotoxic effect in both platinum‐sensitive and platinum‐resistant ovarian cancer cells, rendering them more susceptible to drug‐induced apoptosis [224]. Conversely, strategically enhancing GLUT1 expression in CAR‐T cells has been shown to improve their metabolic fitness and antitumor efficacy within the immunosuppressive TME [122].

### 7.2 Emerging Therapies

A major obstacle to the clinical translation of systemic GLUT1 inhibitors is the unacceptable on‐target toxicity in normal tissues—such as the BBB and erythrocytes—which risks severe neurological side effects (resembling Glut1DS) and hemolysis. To circumvent this, research focus has shifted toward nano‐targeted delivery systems and spatiotemporally controlled regimens.

#### 7.2.1 GLUT1 as a Gateway for Targeted Drug Delivery

Capitalizing on the high‐glucose avidity of cancer cells, nanocarriers are functionalized with glycans to achieve GLUT1‐mediated endocytosis. Strategies include glucosamine‐labeled liposomes capable of “transcytosis” to reach deep hypoxic regions and cancer stem cells [225], N‐acetyl‐d‐glucosamine (GLcNAc)‐functionalized solid lipid nanoparticles (SLNs) loaded with paclitaxel [226], and mannose‐decorated nanomicelles (Man‐NIT) that deplete NADPH and generate ROS [227]. Directly conjugating therapeutics to glucose, such as the glucose–methotrexate conjugate (GLU‐MTX), significantly enhances targeted drug uptake and delays tumor growth while reducing toxicity [228]. More complex nanoplatforms, such as metal‐organic frameworks (MOFs), enable multi‐pronged attacks. For example, ZIF‐8 nanoparticles achieve a dual blockade by releasing Zn^2^^+^ ions to inhibit glycolysis alongside a co‐delivered DNAzyme that cleaves GLUT1 mRNA [229]. Another ZIF‐8 system co‐delivers mitoxantrone (MTX) and thymopentin (TP5) to simultaneously starve the cancer cells and activate the cGAS‐STING pathway for a robust antitumor immune response [230].

#### 7.2.2 Spatiotemporal Control and TME Remodeling

To ensure precise inhibitor release and minimize off‐target effects, spatiotemporal “smart” systems have been designed. These include a photocaged inhibitor (WZB117‐PPG) activated exclusively by visible light [231] and sonodynamic nanoparticles that release BAY‐876 upon ultrasound exposure to induce disulfidptosis in bladder cancer [232]. Advanced delivery formats also facilitate profound TME remodeling. An injectable thermogel providing sustained release of BAY‐876 prevents lactate excretion, reversing the “cold” TME and synergizing with PD‐1/PD‐L1 blockade in glioblastoma [233]. Similarly, delivering BAY‐876 via extracellular vesicles (EVs) specifically reprograms the metabolic state of CAFs, which physically “softens” the dense extracellular matrix and enhances cytotoxic CD8+ T cell infiltration [203].

#### 7.2.3 Indirect Modulation and Targeted Degradation

Emerging strategies also utilize gene and protein manipulation to downregulate GLUT1. A peptide‐based PROTAC degrading the FOXM1 transcription factor concurrently decreases GLUT1 and PD‐L1 expression [234], while mesenchymal stem cell‐derived exosomes delivering miR‐214‐3p indirectly suppress both GLUT1 and ATP citrate lyase (ACLY) [235]. Finally, in a paradigm‐shifting approach, the “Glut1‐facilitated lysosomal degradation” (GFLD) strategy employs antibody‐glycooligomer conjugates to bridge GLUT1 with target membrane proteins such as PD‐L1. This effectively hijacks the GLUT1 transport machinery to traffic pathogenic membrane proteins into the lysosome for degradation, offering a versatile new tool for cancer immunotherapy [236].

### 7.3 Summary of Preclinical Evidence

To date, no therapeutic agents specifically targeting GLUT1 have entered clinical trials, with all current research remaining in the preclinical phase. Table 2 summarizes the primary preclinical inhibitors and their respective therapeutic strategies. Recent patents highlight innovative progress in the field: for example, Southwest Jiaotong University (CN202210700001.1) developed targeted nanoparticles for the co‐delivery of GLUT1 and autophagy inhibitors, significantly enhancing antitumor efficacy through synergistic effects. Additionally, Xiamen Hospital of Traditional Chinese Medicine (CN202011001845.4) demonstrated the protective potential of WZB117 in mitigating liver injury by reducing inflammatory cytokine levels and inhibiting hepatocyte apoptosis. These strategies provide vital references for improving drug efficacy and expanding therapeutic applications.

**TABLE 2:** Summary of GLUT1‐targeting inhibitors and therapeutic strategies in preclinical studies.

Agent name	Target disease	Stage	Institution and country	References
BAY‐876	Tumors	Preclinical	Bayer AG (Germany)	[237]
WZB117	Nervous system diseases	Preclinical	Shanghai Institute of Materia Medica, CAS (China)	[238]
ICO‐33	Pancreatic cancer	Preclinical	Icosagen AS (Estonia)	[239]
STF‐31	Ovarian cancer	Preclinical	Zhejiang University (China)	[240]
PeS‐9	Prostate cancer	Preclinical	Universitätsklinikum Hamburg‐Eppendorf (Germany)	[241]
SRI‐37683	Glioblastoma	Preclinical	The University of Alabama at Birmingham (USA)	[242]
SRI‐37683	Tumor	Preclinical	Zhengzhou University (China)	[243]

## 8 Conclusions and Prospects

In this review, we have sought to outline the multifaceted biology of GLUT1, exploring its transition from a fundamental regulator of physiological homeostasis to a key participant in oncogenic metabolism and tumor microenvironment (TME) remodeling. As the field gradually advances from foundational discoveries toward clinical translation, synthesizing these multidimensional insights may offer a useful perspective. In this concluding section, we reflect on the dual nature of GLUT1, discuss its role in driving metabolic competition within the TME, and explore potential strategies to overcome existing pharmacological challenges, with the hope of providing insights for the future development of precision metabolic immuno‐oncology.

### 8.1 The Dual Nature of GLUT1 in Health and Malignancy

This comprehensive review consolidates the multifaceted biology of glucose transporter 1 (GLUT1), underscoring its profound duality as both an indispensable guardian of physiological homeostasis and a formidable driver of pathology. From an evolutionary perspective, the ubiquitous conservation and high‐affinity kinetics of GLUT1 highlight its nonredundant role in sustaining basal energy requirements, particularly across specialized boundaries such as the BBB [244] and in erythrocytes. The catastrophic consequences of its disruption are starkly illustrated by Glut1DS, where haploinsufficiency precipitates severe neurological energy crises [245]. Conversely, in the oncological landscape, this highly calibrated metabolic gatekeeper is perversely hijacked. It is crucial to recognize that GLUT1 is not an oncogene per se, but rather a highly efficient metabolic facilitator that oncogenic networks (such as MYC, KRAS, and HIF‐1α) exploit to sustain the Warburg effect [246]. Driven by a multidimensional regulatory network encompassing transcriptional hyperactivation and posttranslational stabilization, the pathological upregulation of GLUT1 fuels relentless cellular proliferation. It endows malignant cells with remarkable metabolic plasticity, allowing them to dynamically adapt to severe hypoxic and nutrient‐deprived stressors that would normally trigger apoptosis in nontransformed cells.

### 8.2 Metabolic Competition and TME Remodeling

The clinical significance of GLUT1 extends far beyond satisfying the cell‐autonomous bioenergetic demands of the tumor; it acts as a dynamic architect of the TM. Ubiquitously overexpressed across a wide spectrum of malignancies, elevated GLUT1 levels consistently correlate with aggressive phenotypes, metastatic propensity, and unfavorable patient survival. Notably, by aggressively monopolizing local glucose, GLUT1‐overexpressing tumor cells inflict a state of severe metabolic deprivation on the surrounding stroma, creating a spatial metabolic compartmentalization. This intense nutrient competition effectively strips tumor‐infiltrating lymphocytes (TILs) of the glucose required for their clonal expansion and effector functions.

Furthermore, this metabolic tug‐of‐war initiates a cascade of profound ecological remodeling within the TME. The massive influx of glucose via GLUT1 inevitably leads to the excessive extrusion of lactate, orchestrating a highly acidic and immunosuppressive niche. Recent insights suggest that this lactate‐rich environment not only physically impairs cytotoxic T cells but also chemically alters the epigenetic landscape of stromal cells—such as inducing histone lactylation in TAMs—driving their polarization toward a pro‐tumorigenic M2 phenotype. Concurrently, it metabolically re‐educates CAFs to engage in reverse Warburg metabolic symbiosis, thereby fortifying physical barriers to treatment. Thus, GLUT1 is not merely a nutrient conduit; it is the linchpin that sustains an immunosuppressive ecosystem, driving profound therapeutic resistance and immune evasion.

### 8.3 Overcoming Therapeutic Roadblocks via Precision Medicine

While the central role of GLUT1 in cancer metabolism presents a compelling therapeutic vulnerability, translating this knowledge into clinical practice reveals a classic pharmacological paradox. The primary roadblock has been the unacceptably narrow therapeutic window associated with systemic GLUT1 inhibition. Given the absolute reliance of the brain and erythrocytes on GLUT1, traditional nonselective blockades risk severe neurotoxicity and hemolysis. Moreover, tumors exhibit immense metabolic plasticity, rapidly activating compensatory uptake mechanisms through alternative transporters like GLUT2 or GLUT4 when confronted with direct competitive inhibitors.

Consequently, the therapeutic landscape is undergoing a paradigm shift, moving away from monolithic systemic blockades toward sophisticated, precision manipulations. Rather than fighting GLUT1's high affinity for glucose, emerging breakthroughs ingeniously exploit it as a “Trojan horse.” Glycan‐functionalized nanocarriers use GLUT1 as a highly efficient gateway for targeted drug delivery, achieving deep tumor penetration via transcytosis while sparing normal tissues. Furthermore, precision platforms such as light‐ or ultrasound‐activated nanomicelles offer exquisite spatiotemporal control, releasing inhibitors exclusively within the tumor bed. More revolutionary is the advent of targeted protein degradation technologies, including PROTACs and Glut1‐facilitated lysosomal degradation (GFLD). By internally degrading the receptor or utilizing it to traffic pathogenic membrane proteins (e.g., PD‐L1) into lysosomes, these strategies eradicate the physical scaffolding of the transporter, effectively bypassing the rapid compensatory feedback loops that plague traditional inhibitors. These innovations are transforming a critical metabolic liability into a highly specific and powerful therapeutic asset.

### 8.4 Future Directions and Clinical Translation

Looking forward, unlocking the full clinical potential of GLUT1‐targeted interventions will require a highly integrated, multidisciplinary approach. A critical prerequisite for future clinical trials is the development of robust, multi‐omics predictive biomarkers. Because GLUT1 expression is ubiquitous, identifying patient populations most susceptible to its inhibition will require contextualizing its expression with specific genetic vulnerabilities (e.g., RB1 loss, IDH mutations) and real‐time metabolic imaging (e.g., PET/CT metabolomics). Additionally, exploiting the metabolic symbiosis within the TME holds immense clinical promise. Since GLUT1 drives the formation of “cold,” immunosuppressed tumors, combining precision GLUT1 modulators with immune checkpoint inhibitors (ICIs) represents a highly rational strategy to reverse metabolic immune exclusion and reinvigorate exhausted T cells. Another thrilling frontier lies at the intersection of synthetic biology and metabolism: the metabolic engineering of cellular immunotherapies. By intentionally overexpressing GLUT1 in CAR‐T cells, researchers are “arming” these immune effectors with superior metabolic fitness, preventing activation‐induced exhaustion and enhancing their persistence within nutrient‐starved solid tumors. Ultimately, the trajectory of GLUT1 research exemplifies a maturation in our approach to cancer biology. By shifting the therapeutic objective from the simplistic goal of “starving the tumor” to strategically rewiring the metabolic interactions of the entire TME ecosystem, we can pave the way for a new era of precision metabolic immuno‐oncology—one that maximizes durable therapeutic efficacy while minimizing collateral damage to normal physiological health.

## Author Contributions

Y. T. and Z. C. contributed equally to this work. Y. T. and Z. C. were major contributors to writing the manuscript. Z. C. performed the literature search and data curation. Y. Z. conceived the central idea and provided critical direction for the review. W. H. supervised the project and was responsible for funding acquisition. All authors reviewed and approved the final manuscript.

## Funding

This work was supported by grants from the National Natural Science Foundation of China (82472739 and 82173030 to W. H.) and Zhejiang Cancer Hospital National Natural Science Foundation Cultivation Fund for Postdoctoral Researchers (BH2025055 to Y.T.).

## Ethics Statement

The authors have nothing to report.

## Conflicts of Interest

The authors declare no conflicts of interest.

## Data Availability

The authors have nothing to report.
